# Deciphering organ‐specific chemical changes following insect herbivory in *Populus nigra* using comparative metabolomics

**DOI:** 10.1111/plb.70032

**Published:** 2025-05-28

**Authors:** S. Yepes‐Vivas, M. Popp, M. Reichelt, J. Gershenzon, J.‐P. Schnitzler, S. B. Unsicker

**Affiliations:** ^1^ Department of Biochemistry Max Planck Institute for Chemical Ecology Jena Germany; ^2^ Research Unit Environmental Simulation Helmholtz Zentrum München Oberschleißheim Germany; ^3^ Plant‐Environment‐Interactions Group, Botanical Institute Kiel University Kiel Germany

**Keywords:** Chemodiversity, *Lymantria dispar*, metabolomics, non‐targeted analysis, *Populus nigra*, targeted analysis, tree defence

## Abstract

- This study explores the chemical diversity of plant metabolites in different organs of black poplar (*Populus nigra*), a tree species of considerable ecological and economic importance, to broaden our knowledge of organs other than leaves, especially with regard to herbivore‐induced changes.
- Targeted and non‐targeted metabolite analyses were used to investigate the defence responses of black poplar organs, including leaves, wood, bark, and roots, to aboveground feeding damage by caterpillars of the generalist herbivore *Lymantria dispar*.
- The research revealed that metabolic responses to herbivory are organ‐specific, with a large increase in unique features upon insect damage. Herbivory led to more significant changes in central (primary) metabolites than the targeted specialized (secondary) metabolites measured.
- The study concludes that understanding the complexity of organ‐specific metabolism in black poplar can be very useful for investigating plant–herbivore interactions in this tree species.

## Graphical abstract

This study investigated the organ‐specific metabolic response of black poplar to feeding by generalist *Lymantria dispar* caterpillars using targeted and non‐targeted metabolomics.

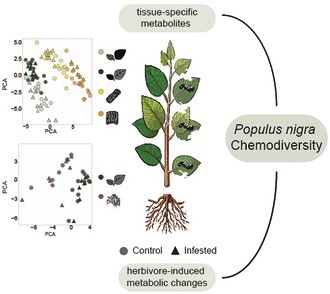

## INTRODUCTION

Plants exhibit greater chemical diversity than most other groups of organisms, synthesizing a remarkable variety of metabolites with different physical and chemical properties (Rambla *et al*. 2015; Leong & Last 2017). Recent estimates suggest that a single plant species can synthesize thousands of metabolites, which can be divided into central and specialized metabolites (Afendi *et al*. 2012; Cope *et al*. 2019). Central metabolites, including sugars, organic acids, and amino acids, are essential for basic cellular functions such as growth, development, storage, and reproduction (Pichersky & Lewinsohn 2011; Yang *et al*. 2020). In contrast, specialized metabolites, also known as secondary metabolites, have an extraordinary structural chemical diversity and play a crucial role in the interaction of plants with their biotic and abiotic environment (Leong & Last 2017; Kessler & Kalske 2018; Maeda 2019; Bertić *et al*. 2021). The biosynthetic pathways of central and specialized metabolites are tightly linked at the metabolite and regulatory levels (Maeda 2019; Zoric *et al*. 2019). The interactions between these pathways provide insights into the overall health and physiological state of plants (Fiehn 2002; Yang *et al*. 2020).

Due to their sessile lifestyle, plants are highly dependent on the accumulation of numerous metabolites in their organs to protect themselves against various biotic or abiotic environmental stresses (Arbona *et al*. 2013; Boeckler *et al*. 2016; Hahn & Maron 2016; Hauri *et al*. 2022). Because the physiology and ecology of plants in their natural environment are so strongly influenced by specialized metabolites, they are sometimes referred to as ‘functional traits’ (Walker *et al*. 2022). These metabolites are highly dynamic and sometimes dramatic metabolic changes are observed in plants interacting with herbivores, pollinators, microbes, or other plants, often strongly depending on the identity of the organism involved (Boeckler *et al*. 2011; Clavijo McCormick, Irmisch, *et al*. 2014; Fabisch *et al*. 2019; Movahedi *et al*. 2021). The dynamic metabolic responses of plants to damage, for example by herbivorous insects, can be explained by metabolic plasticity (Barker *et al*. 2019; Cope *et al*. 2019), and the reallocation of resources within the plant to balance growth and defence trade‐offs (Huot *et al*. 2014).

Studying the complexity and variability of specialized metabolites at different biological scales is of crucial importance if we are to gain a full understanding of the plants´ adaptations to fluctuating environmental conditions. Furthermore, understanding intraspecific metabolic plasticity is essential to explain how plants regulate their metabolic networks in different organs and under different stress conditions. In recent decades, a significant amount of research has been dedicated to investigating the remarkable intra‐ and interspecific chemodiversity observed in plants, and exploring the underlying mechanisms generating this diversity (Wittmann & Bräutigam 2025, Thon *et al*. 2024; reviews by Petrén *et al*. 2024; Wetzel & Whitehead 2020; Kessler & Kalske 2018 and references therein). The majority of studies on chemodiversity have focused on short‐lived herbaceous plants. In contrast, there have been few investigations of chemodiversity in long‐lived woody plants (e.g. Bertić *et al*. 2021; Schweiger *et al*. 2021), and even fewer studies have focused on the chemodiversity of different woody plant organs.

The development of improved mass spectrometry methods, particularly for metabolomics studies, has greatly improved our understanding of plant metabolism (Fang *et al*. 2019; Rodrigues *et al*. 2021; Silva *et al*. 2021). However, due to the high complexity and diversity of plant metabolites, no single analytical method can provide a comprehensive profile of all metabolites in a single plant sample. Historically, metabolomics studies (now called targeted metabolomics) have focused on identifying and quantifying specific compounds to understand their role in a particular physiological process or in resistance to a particular biotic or abiotic stress (Ramos *et al*. 2023). In contrast, non‐targeted metabolomics aims to gather as much information as possible about the metabolites in a sample, whether known or unknown, to characterize their identities through network analysis (Kaling *et al*. 2015; Moritz *et al*. 2017). The combination of both targeted and untargeted approaches can help to obtain a much broader view of the metabolomes of different plant species or plant parts (Rambla *et al*. 2015; Tebbi & Debbache‐Benaida 2022; Shen *et al*. 2023).

Black poplar (*Populus nigra*) is a fast‐growing deciduous tree species native to Eurasia, with considerable economic value in breeding programs to obtain fast growing hybrids for environmental restoration (de Rigo *et al*. 2016), but also for the paper industry and energy cropping. In addition to its economic importance, *P. nigra* acts as a pioneer tree in natural floodplains, contributing to the biodiversity and stability of European ecosystems (Corenblit *et al*. 2014). Black poplar has a large number of specialized metabolites that can be induced by biotic stress, such as insect herbivory and pathogen infestation, and can also provide protection against these antagonists (Boeckler *et al*. 2011; Clavijo McCormick, Boeckler, *et al*. 2014; Fabisch *et al*. 2019; Lackner *et al*. 2019; Ullah *et al*. 2019). However, despite its ecological and economic importance, studies on its metabolome have predominantly focused on characterizing the chemical composition of the leaves (e.g., Boeckler *et al*. 2013) and buds (Stanciauskaite *et al*. 2021), neglecting other important organs, such as the stem and roots, with a few exceptions (Tebbi & Debbache‐Benaida 2022; Li *et al*. 2023). Filling these gaps in our understanding of black poplar metabolism may provide more context for investigating the ecological interactions of this species and thus contribute to its management for purposes of both economics and conservation.

Our study is framed within the concept of metabolic plasticity and resource allocation, which suggests that plants redistribute metabolites in response to stress to optimize resource use. The objective of this study was to investigate the chemodiversity of different organs of young *P. nigra* trees using targeted and non‐targeted metabolomic analyses. The metabolome of different organs was examined, following aboveground herbivory by generalist *Lymantria dispar* caterpillars. This was done in order to understand how metabolic responses are structured across plant organs. We hypothesized that (i) while metabolic profiles *in P. nigra* will vary between organs, they will not be completely distinct, as plants tend to transport key compounds across organs to mitigate stress without incurring excessive energetic costs. Furthermore, we predicted that (ii) herbivory‐induced metabolic changes will be strongest in the damaged organs, but certain metabolites may be relocated to roots or other storage organs in order to maintain metabolic homeostasis.

## MATERIALS AND METHODS

### Plant and insect material and herbivory treatment

#### Stem–leaf comparison

Black poplar (*Populus nigra*) trees for this experiment were propagated from stem cuttings of a single tree genotype cultivated at the Max‐Planck Institute for Chemical Ecology in Jena, Germany. This genotype originally grew in a floodplain forest at the river Odra in northeastern Germany. The rooted stem cuttings were established in 2 L pots filled with pure sand and maintained in a greenhouse under summer conditions (24°C, 60% relative humidity, 16 h light/8 h dark photoperiod) for a duration of 3 months prior to their utilization in the experiment. Subsequently, the trees were acclimated in a climate chamber set at 60% humidity, with day/night temperatures of 20°C/16°C and a 16‐h light period, for 1 day before commencement of the experiment. To assess the impact of spongy moth (*Lymantria dispar*) caterpillar infestation on the metabolic changes in black poplar, we performed a comparative analysis between non‐infested trees (*n* = 12) and trees subjected to caterpillar infestation (*n* = 12). We chose the spongy moth, a generalist herbivore and a natural enemy of black poplar in Europe (Lindroth 1991), to induce herbivory stress because of its high ecological relevance and wide host range. Caterpillars, hatched from eggs (kindly provided by Hannah Nadel, USDA) were reared on an artificial wheatgerm diet (MP Biomedical, Eschwege, Germany) in a climate chamber maintained at 25 °C and 60% humidity, with a 14‐h day/10‐h night cycle, until they reached the 3^rd^ to 4^th^ instar. Prior to the experiment, 96 caterpillars were individually placed in plastic cups (Solo Cup, Highland Park, IL, USA), weighed, and subjected to a 24‐h starvation period. Eight 3^rd^ to 4^th^ instar caterpillars were released onto each designated “*infested tree*” and allowed to feed for a period of 48 h. Caterpillars were enclosed on the trees with a mesh and placed in a climate chamber with conditions set at 25°C, 60% humidity, and a 14‐h day/10‐h night cycle. After 48 h, the caterpillars were removed, and samples of leaves, bark, and wood were collected from each tree.

Although roots were collected during this experiment, they accidentally thawed during processing and thus could not be further analysed. To account for root metabolome analysis, we used data from a previous experiment (*leaf–root comparison*) conducted using a second black poplar genotype and the same caterpillar species. To ensure the metabolic consistency between experiments, we performed a Principal Components Analysis (PCA) on the non‐targeted leaf data, which showed that the leaves from both experiments clustered together, confirming the validity of using these datasets (Fig. S1). The other plant organs formed distinct clusters. The comparison was thus conducted while considering the differences between the experiments. Details on the software, data scaling, and transformation used for the PCA are provided in the statistical analysis section below.

#### Leaf–root comparison

This experiment was conducted using a second genotype of *P. nigra* originating from the same natural population as mentioned above. Here, 30 trees were reared from monoclonal stem cuttings in the greenhouse for a duration of 4 months before being acclimatized in a climate chamber under the same conditions as described above (Lackner *et al*. 2019). Due to space limitations, the experiment was divided into three blocks, with each block consisting of 10 trees and an equal number of replicates for each treatment. The time between the start of the experiments in block 1 and block 3 was 4 weeks, with the second block starting approximately 2 weeks after the first block. Spongy moth caterpillars were reared as described above. To assess the effect of spongy moth caterpillar infestation on metabolic changes in leaves and roots of black poplar, a comparative analysis was made between non‐infested trees (*n* = 15) and those subjected to caterpillar infestation (*n* = 15). Six fourth instar caterpillars were released on each designated ‘infested tree’ and allowed to feed for 40 h. After this time, the caterpillars were removed and samples of leaves and roots collected and pooled per individual tree.

### Plant organ harvest and sample preparation

After removing the caterpillars, tree height was measured, healthy and damaged leaves in each tree were counted and weighed. The first five fully developed leaves from the apex in the basipetal direction were identified as young, while the next six leaves downward from the apex to the base were categorized as old. For the *stem–leaf comparison*, these were kept separately, but for the *leaf–root comparison* they were combined. Three young and five old leaves were cut from the stem and arranged on a whiteboard for photographic documentation using a digital camera. The midvein was then removed from each leaf with scissors, and leaf halves were placed in 5 mL tubes that were immediately flash‐frozen in liquid nitrogen. All remaining leaves on each tree were then also removed, and 3 cm of the stem was carefully cut about 10 cm below the first sampled leaf. The bark of these stem cuttings was then peeled off with a razor blade. Then bark and wood were transferred into 5 mL tubes and immediately flash‐frozen in liquid nitrogen. After the soil was shaken off the roots, the roots were carefully cleaned under a waterjet. The roots of each tree individual were placed in 50 mL Falcon tubes before they were also flash‐frozen in liquid nitrogen. All samples were stored at −80°C until all plant organs were lyophilized (Alpha 1–4 LD plus; Martin Christ, Osterode, Germany). Afterwards, all samples were ground to fine powder by agitating them in cooled vessels with metal beads for 2 min at 60 rpm. An aliquot of the powder was then sent via post from Munich to Jena for targeted metabolic analyses.

### Leaf herbivory measurements

The total leaf area and the portion of missing leaf area resulting from spongy moth feeding was determined, employing the approach outlined in Boeckler *et al*. (2013). In essence, the total sum of missing and present leaf area served as the denominator to calculate the percentage of herbivory. This was achieved by dividing the pixel count of the absent leaf area by the total missing + present leaf area, and the resulting quotient was then multiplied by 100. For the conversion of pixel numbers to actual area measurements in square centimetres, a reference square (black 2 × 2 cm^2^) on the whiteboard was used as a scale. The analysis of both present and missing leaf areas was conducted using Adobe Photoshop 24.4.1.

### Non‐targeted LC–MS measurement

Sample preparation for the non‐targeted analysis was done using a Hamilton robot (Hamilton, Bonaduz, Switzerland) and followed the protocol developed by Bertić *et al*. (2023). An aliquor of 25 mg of freeze‐dried plant material was mixed with 800 μL methanol/isopropanol/water (1/1/1) (Merck, Darmstadt, Germany). The solvent contained a mixture of non‐plant origin compounds, which served as internal standard. A list of the standards used can be found in Table S1. After mixing and ultrasonication for 30 min, the samples were blown dry under nitrogen, and 800 μL acetonitrile/water (1/1) (Merck) were added to each sample. The samples were again put in the ultrasonication bath for 30 min, and after centrifugation at 200 rpm, the supernatants were used for further chemical analysis performed on an ultra‐performance liquid chromatograph (UPLC) ultra‐high resolution (UHR) tandem quadrupole/time‐of‐flight (QqToF) mass spectrometer (MS) (UPLC: Ultimate 3000RS UPLC from ThermoFisher Scientific, Bremen, Germany; MS: Bruker Impact II (QqToF) and an Apollo II electrospray ionization (ESI) source) using an reverse‐phase column (C18, Acquity BEH, Waters, Eschborn, Germany, 150 mm × 2.1 mm, 1.7 μm). For compound elution, we used solvent A (0.1% formic acid in water) and solvent B (0.1% formic acid in acetonitrile). Gradient: 0–1 min, 95% A (isocratic); 1–15 min, 95%–70% A, 15–17 min, 70%–20% A, 17–20 min 20% A (isocratic), 20–22 min 20%–0.5% A, 22–27 min 0.5% A (isocratic), 27–29 min 0.5%–95%A, and 29–31 min 95% A (isocratic). The constant solvent flow was 0.4 mL min^−1^, and the column temperature was 40°C. We injected 5 μL of the sample. Mass calibration was performed with the calibration 1% NaOH in water/isopropanol (1/1). We measured both positive (+) and negative (−) ionization modes. The nebulizer pressure was 2.0 bar, dry gas flow was 8.0 L min^−1^, dry gas temperature was 200°C, capillary voltage was 4500 V for (+) and 3500 V for (−); endplate offset was 500 V; mass range was 20–2000 *m/z*. The chemicals were LC–MS hyper‐grade and were all purchased from Merck, except 2‐propanol and acetonitrile, which was purchased from Honeywell (Puchheim, Germany).

Afterwards, the mass features obtained from both modes were combined into mass buckets using Metaboscape software (Bruker Daltonics, Bremen, Germany). Peak detection was carried out with the T‐Rex 3D algorithm for qTOF data. For peak detection, the following parameters were used: intensity threshold of 1500 with a minimum of eight spectra, time window from 0.5 to 29.0 min, peaks were kept if they were detected in at least 10% of all replicates of one sample group. EIC correlation to combine common ions (Negative mode: [M−H]−, [M+Cl]−, [M−H2O+H]−, [2M+H]−, [M+CH3CN−H]−, [M+HCOOH−H]−) was 0.7.

The annotation was done on level 3 (according to Sumner *et al*. 2007) using Metaboscapes's smart formula annotation algorithm, which assigns a tentative sum formula using the molecular weight of the feature and the ratio of the elements in the compound (Kind & Fiehn 2007). Using multidimensional stoichiometric compound classification (MSCC; Rivas‐Ubach *et al*. 2018), the features were grouped with high confidence into five primary categories: lipids, proteins, amino sugars, carbohydrates, and phytochemicals (characterized by molecular formulas typical of well‐known (specialized) plant metabolites i.e., alkaloids, phenolics, and terpenoids), based on an evaluation of the C/H/O/N/P stoichiometric ratios. In particular, we distinguished between amino sugars and carbohydrates, as amino sugars contained nitrogen, which allowed us to classify them separately. While nucleotides were part of the possible compound categories in the MSCC framework, they were not detected in our dataset (Wang *et al*. 2016). After normalization by IS mixture (Sysi‐Aho *et al*. 2007), composed of plant metabolites that spread over a wide mass and RT range but are not found in poplar leaves (Table S1) and a performed batch correction, the list of specific masses and the primary category assigned by MSCC and used for statistical analysis.

### Targeted LC–MS and HPLC‐UV measurements

For the targeted analysis, 10 mg freeze‐dried plant material was extracted with 1 mL 100% MeOH containing 0.8 mg phenyl‐β‐glucopyranoside (Sigma‐Aldrich, Deisenhofen, Germany) and phytohormone standards (40 ng D4‐SA (Santa Cruz Biotechnology, TX, USA), 40 ng D6‐JA (HPC Standards, Germany), 40 ng D6‐ABA (Toronto Research Chemicals, Toronto, Canada), and 8 ng D6‐JA‐Ile (HPC Standards)). Trifluoromethyl cinnamic acid (Alfa Aesar, Thermo Scientific) was added at a concentration of 10 ng mL^−1^, and syringic acid (Sigma Aldrich) served as internal standard at a concentration of 100 ng mL^−1^. The samples were agitated on a mixer and for 30 min at 200 rpm on a horizontal shaker (IKA Labortechnik, Staufen im Breisgau, Germany). Following centrifugation for 5 min at 3200 rpm, the supernatants were employed for analysis of amino acids, sugars, and phenolics.

For salicinoid analysis, 400 μL of the initial methanol extract was combined with 400 μL Milli‐Q H_2_O, and salicinoids measured using high‐performance liquid chromatography‐ultraviolet detection (HPLC‐UV; Agilent 1100 HPLC system, Agilent Technologies, Waldbronn, Germany), following the method outlined in Boeckler *et al*. (2013). Subsequently, 10 μL of the diluted extract was injected into a reverse‐phase chromatographic column (EC 250 × 4.6 mm NUCLEODUR Sphinx RP, 5 μm, Macherey Nagel, Düren, Germany) connected to a pre‐column (C18, 5 μm, 4 × 3 mm, Phenomenex, Aschaffenburg, Germany). The mobile phases, solvent A (Milli‐Q H_2_O) and solvent B (acetonitrile), were run in gradient mode. The gradient time (min)/concentration of B (%) was set to 0/14; 22.00/58; 22.10/100; 25.00/100; 25.10/14; 30.00/14 with a constant flow rate of 1 mL min^−1^. The column oven temperature was set at 25°C. Detection occurred at 200 nm with a photo diode array detector (PDA, Agilent 1100 DAD). Retention times (RT) for the compounds were 5.2 min (salicin), 6.3 min (PAB1), 7.4 min (catechin), 10.3 min (salicortin), 15.2 min (homaloside D), and 19.3 min (salicortin‐6‐benzoate). Concentrations of phenolic compounds were determined relative to the internal standard of phenyl‐β‐glucopyranoside using the following response factors (0.2 PAB1, 0.3 catechin, 0.4 salicin, 0.6 homaloside D and salicortin‐6‐benzoate, and 0.9 salicortin).

For free amino acid analysis, the raw extracts underwent a 1:10 dilution with water containing an isotopically labelled amino acid mix (^13^C), ^15^N‐labelled amino acid mix at a concentration of 10 μg of the mix per mL (Isotec, Miamisburg, OH, USA) and 5 μg mL^−1^ D5‐tryptophan (Cambridge Isotope Laboratories, Andover, MA, USA). Subsequently, the extracts were subjected to measurement using HPLC (Agilent 1260 series HPLC system coupled to a triple quadrupole mass spectrometer) QTRAP6500 (AB Sciex, Darmstadt, Germany). Separation was achieved on a Zorbax Eclipse XDBC18 column (50 mm × 4.6 mm, 1.8 μm; Agilent). The mobile phases included solvent A (0.05% formic acid in water (v:v)) and solvent B (acetonitrile). The chromatographic gradient was set as follows (time in min/concentration of solvent B in %): 0.00/3, 1.00/3, 2.70/100, 3.00/100, 3.10/3, 6.00/3. The constant flow rate was 1100 μL min^−1^, and the column oven temperature was maintained at 25°C. Ionization occurred in positive electrospray ionization mode. Multiple reaction monitoring (MRM) was employed to track analyte parent ion to product ion, with MRMs selected as in Jander *et al*. (2004), as indicated in Table S2. Data acquisition utilized Analyst 1.6 software (AB Sciex), while MultiQuant 3.0.3 software (AB Sciex) facilitated processing. Individual amino acids in the sample were quantified using their respective ^13^C, ^15^N‐labelled amino acid internal standards.

For analysis of soluble sugars, raw extracts of the samples were diluted 1:10 (v:v) in water containing 5 μg mL^−1 13^C6‐glucose (Sigma‐Aldrich) and 5 μg mL^−1 13^C6‐fructose (Toronto Research Chemicals). Glucose, fructose, sucrose, mannitol, raffinose, and stachyose were quantified using an Agilent 1200 series HPLC system (Agilent Technologies) coupled to an API 3200 mass spectrometer (AB Sciex), following the protocol described in Madsen *et al*. (2015). Separation occurred on an apHeraTM NH2 polymer HPLC column (15 cm × 4.6 mm, 5 μm; Supelco) using Milli‐Q H_2_O (A) and acetonitrile (B) at a flow rate of 1.0 mL min^−1^, with an elution profile as follows (time in min/concentration of solvent B in %): 0.00/80, 0.50/80, 13.00/55, 14.00/55, 14.10/80, 18.00/80. Electrospray ionization (ESI) in negative ionization mode facilitated the coupling of LC to MS. The mass spectrometer parameters were configured as follows: ion spray voltage, −4500 V; turbo gas temperature, 600°C; collision gas, 5 psi; curtain gas, 20 psi; ion source gas 1, 50 psi; ion source gas 2, 60 psi. Multiple reaction monitoring (MRM) tracked analyte parent ion to product ion formation (Table S3). Data acquisition utilized Analyst 1.5.1 software, and quantification was conducted using MultiQuant 3.0.3 software (Sciex). Concentrations of glucose and fructose were determined relative to the internal standards of ^13^C6‐glucose and ^13^C6‐fructose, respectively, while the contents of sucrose (Sigma‐Aldrich), mannitol (Fluka, Deisenhofen, Germany), raffinose, verbascose, and stachyose were calculated based on external standard curves.

### Statistical analysis

All data were checked for statistical assumptions, including normal distribution and homogeneity of variances. Data acquired by targeted analysis were normalized with Winsorization mean transformation using the R package “*robustbase*”. After normalization, a Linear Mixed Effects Model (LMER) was performed using the R package “*lmer*” on the data for amino acids, sugars and phenolic compounds, both before and after exposure to herbivores (Tables S4–S6). Tree identity was used as random effect, and the fixed effects were organ, treatment, and the interaction between these two terms. A log transformation was applied to the heat map of compounds of interest. Detailed statistical information is provided in the figures, including mean ± SE and significance levels (*: *P* < 0.05; **: *P* < 0.01; ***: *P* < 0.001).

Data acquired by non‐targeted analysis were normalized to the internal standard and the background was subtracted. Before conducting multivariate statistical analysis, the non‐targeted data were log‐transformed (log10), centred, and (Pareto)‐scaled following established protocols (Van den Berg *et al*. 2006; Eriksson *et al*. 2013). Utilizing SimcaP (v. 13, Sartorius, Göttingen, Germany), (Two‐way) Orthogonal Partial Least‐Squares Discriminant Analysis (O2PLS‐DA/OPLS‐DA) and PCA models were generated, incorporating a discriminating variable codex of the organ and/or treatment.

In our study, we used the Hill diversity approach to assess the chemodiversity of *P. nigra* for non‐targeted data. Hill diversity provides a concise quantitative assessment of compound richness and evenness, considering both the number of unique peaks and their relative abundances (Chao *et al*. 2014). Hill‐Shannon diversity was calculated at *q* = 1, which incorporates both richness and evenness, being the exponential of Shannon Diversity (Petrén *et al*. 2024). The Hill‐Shannon diversity calculations for non‐targeted data were performed utilizing the R‐package *chemodiv* (Petrén *et al*. 2023). The data were normalized using a log transformation. Following normalization, LMER analysis was conducted on the Hill‐Shannon Diversity index using the R package “*lmer”* (Table S7). A post‐hoc analysis using the R package “*emmeans*” was then performed to assess pairwise comparisons between organs and treatments.

For the volcano plots, the *P*‐values and the log_2_ fold change were calculated for each organ comparing control and treatment. All analyses were run in R (RStudio v. 4.2.1) using the package *ggplot2* for graphical visualization.

## RESULTS

### Organs differ in metabolite composition

Using both targeted and non‐targeted metabolomics, the chemical composition of *Populus nigra* saplings was analysed from five different organs in two experiments: a *stem–leaf comparison* (young leaves vs. old leaves vs. bark vs. wood) and a *leaf–root comparison* (leaves vs. roots).

**Fig. 1:**
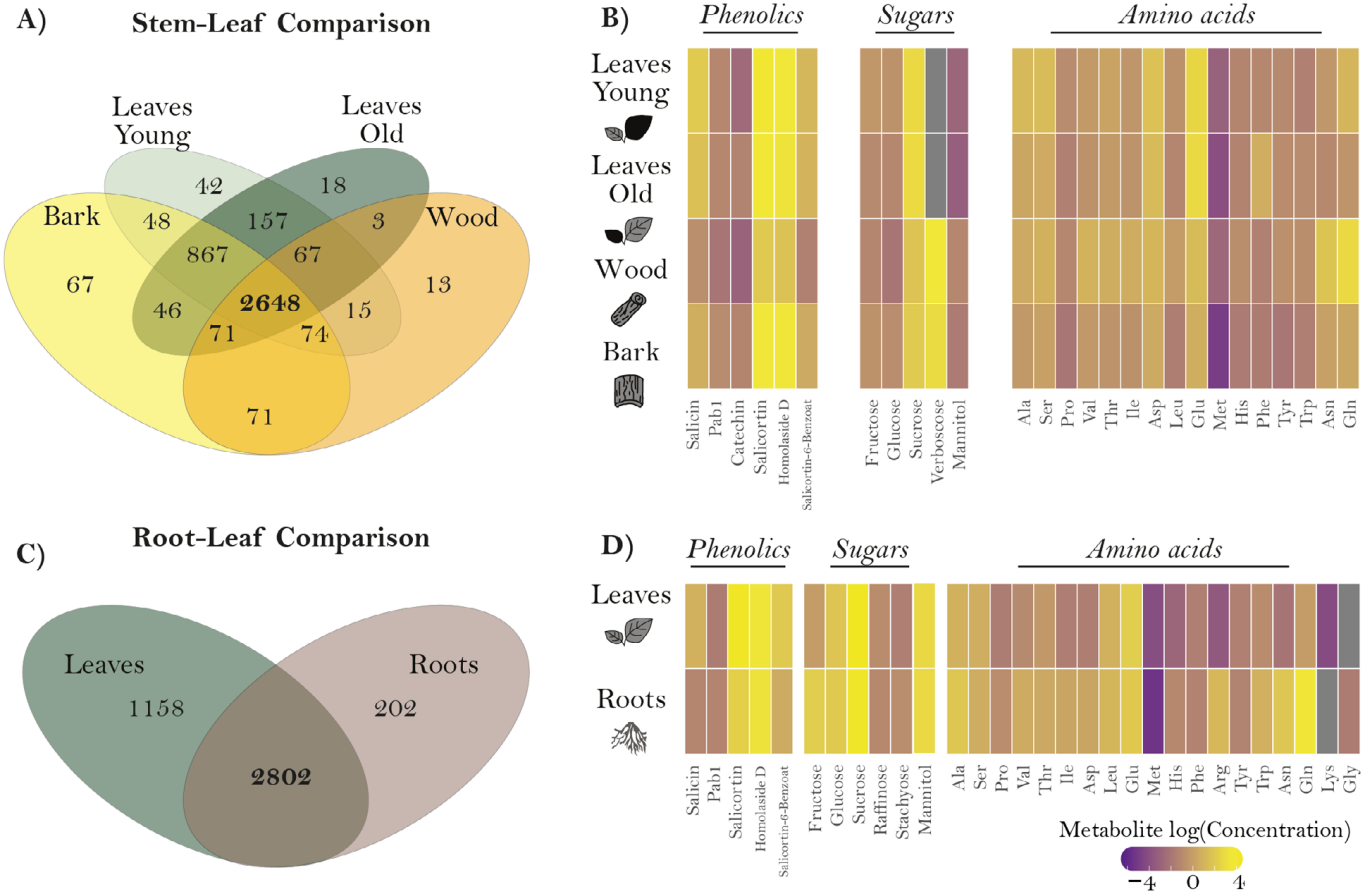
Metabolite comparison in different organs of black poplar. Venn diagrams illustrate the numbers of features identified in the non‐targeted analysis for the *stem–leaf* (*n* = 12) (A) and the *root–leaf comparison* (*n* = 15) (C). In the Venn diagrams, the overlapping regions represent the shared metabolic features between different organs. Heat map representation of the compounds detected in the targeted analysis for both the *stem–leaf* (B) and the *root–leaf comparison* (D). Colour indicates the relative abundance (log) of metabolites. Grey colour indicated the absence of the compound in the analysed organ. Missing compounds in *stem–leaf comparison* were not detected in the targeted analysis.

For the non‐targeted metabolomics data, we first checked for shared *m*/*z* features between organs, defining an *m*/*z* feature to be present in an organ when it had a value other than zero. The resulting *m/z* features for each organ were visually represented using a Venn diagram (Fig. 1A,C). A total of 4207 features were found in the stem, wood, and leaves (Fig. 1A). Notably, 2648 features were found to be common to all organs, representing 63% of the selected features. Surprisingly, the organs that were expected to share the most features due to their similarity in function i.e., young and old leaves, wood and bark, shared only 157 and 71 features, respectively, which were not shared between other organs. Only 5.3% of the selected features were unique to a specific tissue, including 42 for young leaves, 18 for old leaves, 67 for bark, and 13 for wood. For the *root–leaf comparison* (Fig. 1C) 4162 features were present. Unexpectedly, leaves and roots shared 2802 of the features, representing 67% of the total. Interestingly, leaves had four times more unique features than roots, with 1158 and 202 features, respectively.

In the targeted analysis, 31 metabolites were quantified, including 16 to 19 amino acids, five to six sugars, and the phenolic defence compounds: salicin, salicortin, salicortin‐6‐benzoate, homaloside D, proanthocyanidin B1 (PAB1), and catechin (Fig. 1B,D). The heat map illustrates the differences in compound concentration according to the organ examined. In the comparison between the stem and leaves, the composition of phenolic compounds and amino acids showed the greatest differences (Fig. 1B). Similarly, the comparison between roots and leaves revealed differences in most of the compounds analysed, with the exception of a few amino acids and sugars (Fig. 1D). To identify specific patterns, we present the differences in the composition of phenolic compounds (Fig. S3) and sugars (Fig. S4) for both comparisons. The concentration of phenolic compounds was higher in young leaves compared to other organs in the *stem–leaf comparison*, except for catechin, which showed a higher concentration in old leaves (Fig. S3F). Sucrose‐derived sugars, such as raffinose and stachyose, were more abundant in the bark and old leaves (Fig. S4E,F). In contrast, the monosaccharides fructose and glucose, and the disaccharide sucrose were more abundant in leaves than in other organs (Fig. S4A,B,D). When comparing roots and leaves, the concentration of phenolic compounds was higher in leaves, except for PAB1, which had a higher concentration in the roots (Fig. S3D). Concerning sugars, a higher concentration of monosaccharides was observed in the roots (Fig. S4A,B). In contrast, the di‐ and oligosaccharides analysed had a higher concentration in the leaves (Fig. S4C,D).

### Organs differ in chemical diversity

**Fig. 2:**
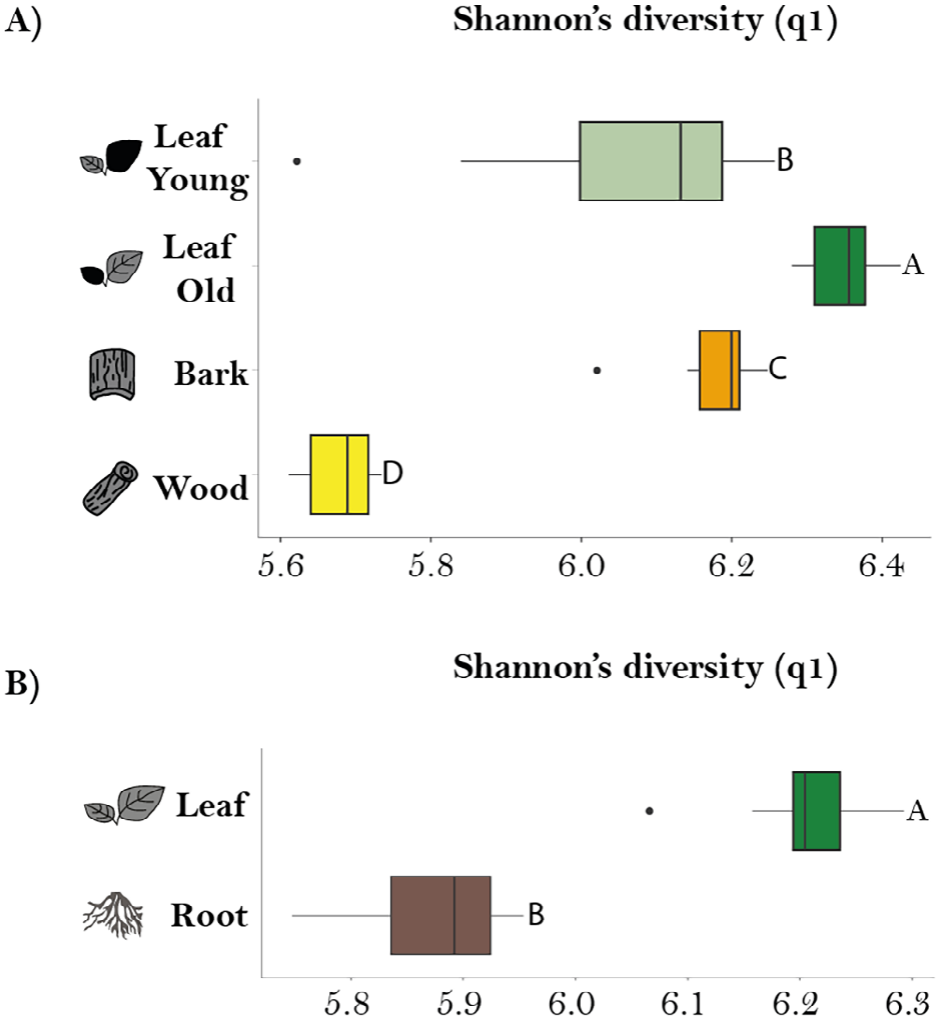
Hill‐Diversity on two different comparisons. Boxplots showing Hill Shannon's diversity calculated from the non‐targeted data for the *stem–leaf* (*n* = 12) (A) and *root–leaf comparison* (*n* = 15) (B). The *x*‐axis denotes Shannon's diversity, and the *y*‐axis lists the organs under investigation. Each boxplot incorporates the median line, interquartile range (IQR), and whiskers extending to 1.5 times the IQR. Individual data points beyond this range are considered outliers and displayed as black dots. Statistically significant differences were determined using a Linear Mixed Effect Model (LMER) (Table S7) followed by a post‐hoc pairwise comparison test between organs.

After observing organ‐specific differences in metabolites, we next investigated whether there were differences in chemodiversity among different *P. nigra* organs by calculating the Shannon diversity index for the non‐targeted metabolomics data (Fig. 2A,B). Hill‐Shannon diversity was highest in old leaves, followed by bark, young leaves and wood for the stem/leaf comparison (Fig. 2A). All organs showed a narrow IQR except young leaves, suggesting less variability within each organ. Statistically significant differences were observed between all of these organs according to the pairwise comparisons conducted using the estimated marginal means (EMMs) test. When comparing leaves and roots, Hill Shannon diversity was higher in leaves in comparison to roots (Fig. 2B). In addition, roots had a relatively wide IQR, indicating a range of diversity values, whereas leaves had a narrow IQR, indicating consistency. The differences between leaves and roots were statistically significant (*P* ≤ 0.001).

### Organs have distinct metabolomes

**Fig. 3:**
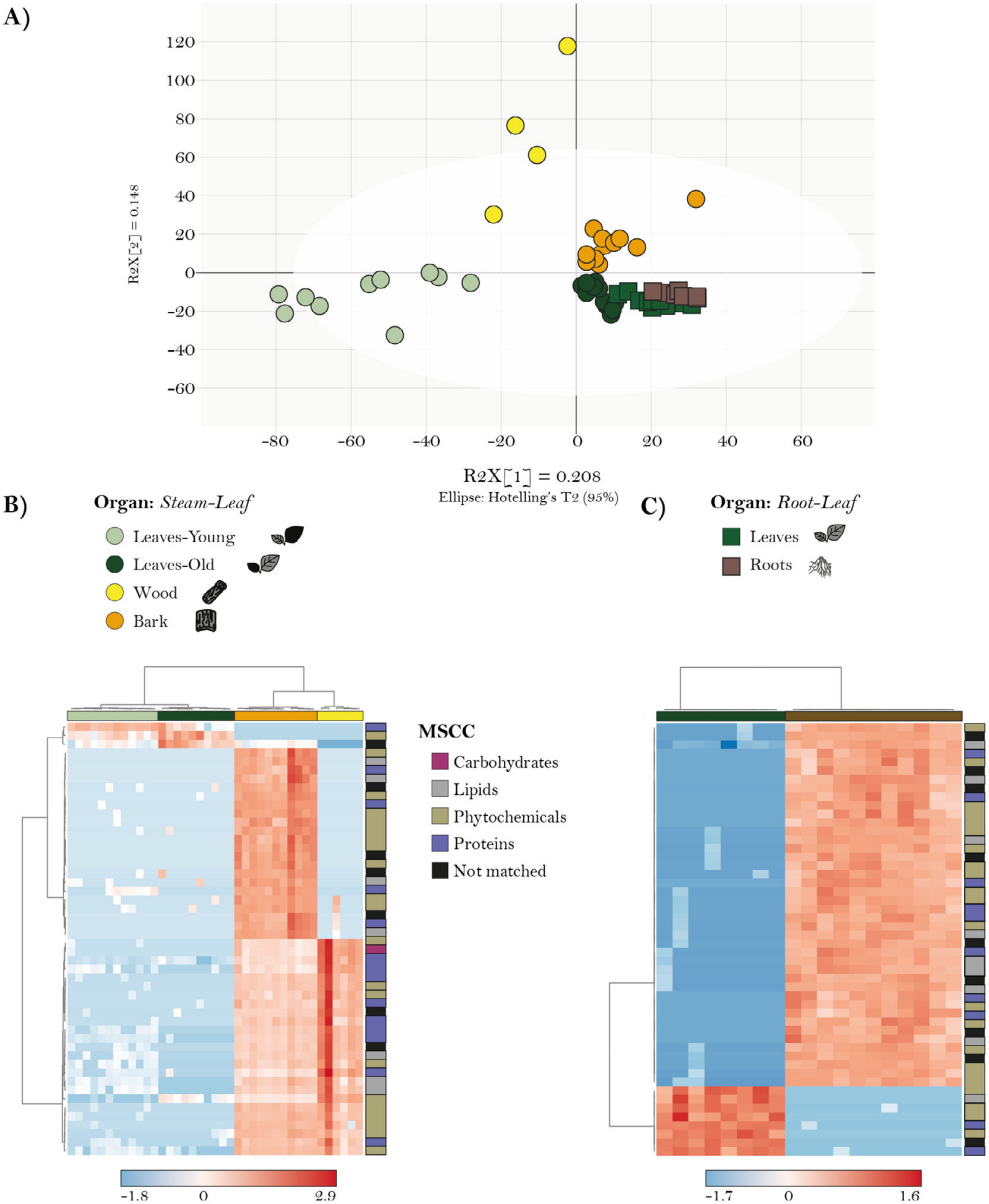
Statistical analysis of non‐targeted metabolite measurements of organs from *stem–leaf* (*n* = 12) and *root–leaf comparisons* (*n* = 15). (A) Orthogonal Projections to Latent Structures Discriminant Analysis (OPLS‐DA) plot showing the separation of samples based on metabolite profiles, grouped by comparison (shape) and organ (colour). The *x*‐axis represents the predictive component (*t*[1], explaining R^2^X[1] = 0.208 of the variation), while the *y*‐axis represents the orthogonal component (*t*[2], explaining *R* ^2^X[2] = 0.148 of the variation). The white ellipse corresponds to Hotelling's *T* ^2^ 95% confidence interval, indicating the multivariate normal range for sample grouping. (B) Hierarchical clustering analysis (HCA) of metabolite features classified using multidimensional stoichiometric compound classification (MSCC) for the *stem–leaf comparison*. (C) HCA for the *root–leaf comparison*. Colours represent different compound classes and organs.

The chemical compositions of the organs investigated could be distinguished based on the Orthogonal partial least squares discriminant analysis (OPLS‐DA) (Fig. 3A). It is notable that old leaves from the *stem–leaf comparison* clustered together with the leaf from the *root–leaf comparison*, validating the comparison between these two experiments. The resulting heat map also illustrated distinct organ clusters (Fig. 3B). Interestingly, bark had the highest relative abundance of compounds across all classes, forming a cohesive cluster with a few exceptions. Wood and leaves also showed distinct clusters, indicating their unique chemical composition. For the *root–leaf comparison* (Fig. 3C), two clear clusters for each organ can be seen, and therefore, an excellent separation of these two organs.

### Herbivory leads to organ‐specific changes in metabolome composition

Organ‐specific metabolic changes in response to insect herbivory were investigated after leaf chewing damage by caterpillars of the generalist spongy moth *Lymantria dispar*. Caterpillars consumed young and old leaves equally (Fig. S2; *P* = 0.13). In the *stem–leaf comparison*, the total number of common *m/z* features across all organs, visualized with a Venn diagram, increased slightly in herbivory‐exposed plants (2881 features; Fig. S5A) compared to control plants without herbivory (2648 features; Fig. 1A). However, the number of unique features in young leaves and bark decreased under herbivory, within control plants exhibiting 67 unique features in bark and 42 in young leaves (Fig. 1A), whereas herbivory‐treated plants showed 42 unique features in bark and 31 in young leaves (Fig. S5A). In contrast, unique features in old leaves and wood increased under herbivory, with old leaves gaining 15 additional features and wood gaining two additional features (Fig. S5A). We further examined the changes in the number of *m/z* features for each organ separately between herbivory‐damaged and control treatments, using a Venn diagram (Fig. S5C). Among all organs, wood was the only organ in which control plants had more unique features than herbivory‐treated plants. For the root–stem comparison, herbivory led to a decrease in the number of shared features across all organs. For example, leaves and roots in control plants shared 2802 features (Fig. 1C), whereas herbivory‐treated plants shared 2657 features (Fig. S5B). Both organs also displayed an increase in unique features, with leaves showing 62 additional unique features and roots 85 (Fig. S5B). When we compared the number of *m/z* features in each organ individually between herbivore‐damaged and control treatments, using a Venn diagram (Fig. S5D), we observed that both leaves and roots in herbivory‐treated plants had a higher number of unique features.

**Fig. 4:**
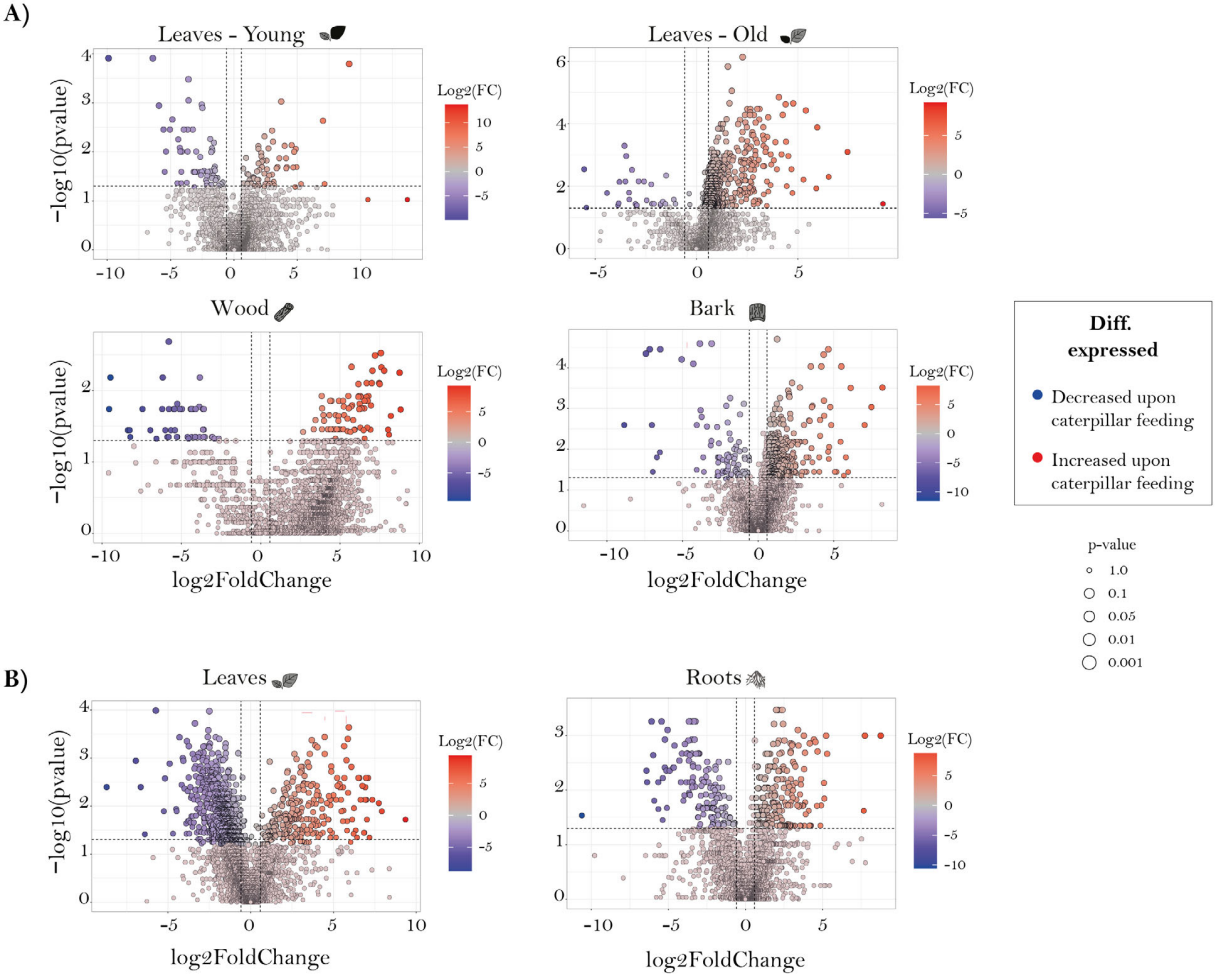
Herbivory‐induced changes in *P nigra* metabolomes of different above‐ and belowground organs. Volcano plots compare features from non‐targeted analysis showcasing alterations in abundance in the presence of herbivory for each organ studied in the *stem–leaf comparison* (*n* = 12 per treatment) (A) and the *root–leaf comparison* (*n* = 15 per treatment) (B). Upward shifts (red dots) represent induction upon caterpillar feeding, while downward shifts (blue dots) indicate induction in control plants in the metabolic features. Black dots do not significantly change upon the treatment. The *x*‐axis represents the log_2_ fold change in relative abundance, while the *y*‐axis represents the −log10 *P*‐value. The horizontal dashed line indicates the significance threshold (*P* value = 0.05), and the vertical dashed lines indicate the fold change cutoffs (log_2_ fold change = ±1.5). Features above the horizontal line and beyond the vertical lines are considered significantly differentially expressed.

To visualize changes in the abundance of metabolic features after herbivory compared to undamaged control plants for each organ, volcano plots were used (Fig. 4). In general, most metabolic features were more abundant in organs of herbivore‐damaged trees except for young leaves in the *stem–leaf comparison* and leaves in the *root–stem comparison* (Fig. 4A,B).

**Fig. 5:**
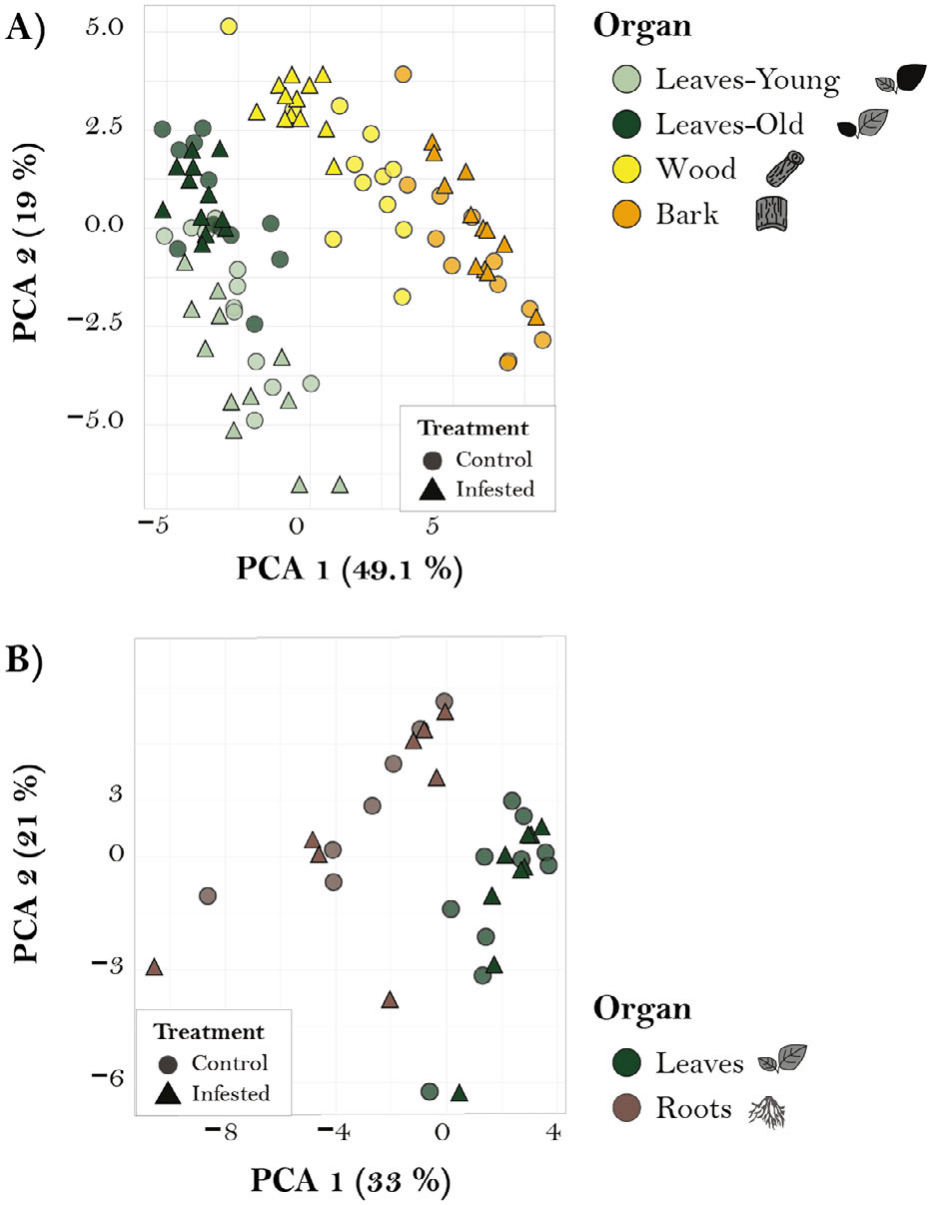
Organ‐specific metabolite profiles obtained from targeted analyses. PCA of compounds measured in targeted analyses, showing the grouping of the samples by organ (colour) and treatment (shape). For the *stem–leaf comparison* PC1 explains 49.1% of the variation, and PC2 explains 19% (A). For the *root–leaf comparison* PC1 explains 33% of the variation, and PC2 explains 21% (B).

Targeted analyses were also conducted to compare the response to caterpillar infestation in various organs. PCA revealed organ grouping in both comparisons but did not show a clear distinction between herbivory and control groups for either comparison (Fig. 5A,B). Similarly, when concentration changes were evaluated using volcano plots, only a limited number of compounds showed discernible up‐ or downregulation (Fig. S6). To identify specific differences in concentrations of analysed compounds across organs and treatments, we conducted a linear mixed effects model (Tables S4–S6). In the *root–leaf comparison*, caterpillar herbivory did not affect any of the phenolic compounds measured (Fig. S3, Table S4) but significantly increased raffinose and stachyose concentrations, while the other sugars were not affected (Fig. S4, Table S5). Caterpillar herbivory also had no effect on amino acid concentrations of different organs, except for tryptophane (Table S6). However, in the *stem–leaf comparison*, both organ and treatment effects were significant for certain compounds. For example, among sugars, treatment significantly affected the concentration of sucrose, increasing its levels in bark and wood, and verbascose, which showed higher concentrations in infested plants. Significant organ–treatment interactions were observed for fructose, sucrose, mannitol, and verbascose (Fig. S4, Table S5). Similarly, the concentrations of several amino acids were significantly influenced by the caterpillar treatment (Table S6), and there were also significant organ–treatment interactions in amino acid concentrations (Table S6).

The differences in chemodiversity among organs in the presence or absence of herbivores were assessed using Hill‐Shannon diversity for both comparisons (Fig. S7). Consistent with previous findings on the metabolome of undamaged poplar, old leaves exhibited the highest Hill‐Shannon diversity, followed by bark and young leaves, and wood in the *stem–leaf comparison*. After herbivory, Hill‐Shannon diversity of the bark decreased markedly compared to controls for bark (Fig. S7A) but increased for wood and young leaves, and no changes in old leaves. In the *root–leaf comparison*, we again observed a higher Hill‐Shannon diversity in leaves compared to roots. After herbivory, we noted an increase in Hill‐Shannon diversity compared to undamaged controls for leaves (Fig. S7B).

## DISCUSSION

Understanding the patterns of chemical diversity within and among plants can make an important contribution towards knowledge of the physiological and ecological functions of specific metabolites. In this study on black poplar (*Populus nigra*), both targeted and non‐targeted metabolomic analyses were performed to determine how chemical composition varies among different organs and how herbivory by spongy moth (*Lymantria dispar*) caterpillars leads to changes in the metabolome. The non‐targeted analyses provided a comprehensive overview of the chemical classes and chemical diversity of different samples, while the targeted analysis permitted precise quantification of a number of specific metabolites to highlight organ‐specific variation.

Within a tree, chemical composition varies depending on factors such as organ, age, season, and environmental conditions (Barton & Boege 2017; Albert *et al*. 2018; Lavola *et al*. 2018; Nissinen *et al*. 2018; Thon *et al*. 2024). Our study in young black poplar trees revealed distinct chemical profiles across the various organs sampled: young and old leaves, bark, wood, and roots. This organ‐specific variation supports our first hypothesis, which proposed that metabolic profiles would vary between organs, but would not be completely distinct. Leaves had the most unique chemical features, and the old leaves showed a more even distribution of metabolic traits and higher alpha diversity compared to young leaves. Meanwhile, bark and wood exhibited a higher frequency of features that were unique to them, accompanied by a lower alpha diversity, indicating a rather unequal distribution of features in wood and bark as compared to other organs investigated. The comprehensive metabolite composition of bark and wood remains relatively understudied (Abreu *et al*. 2020; Huang *et al*. 2021; Li *et al*. 2023). Bark serves a protective function, shielding underlying organs from herbivores, pathogens, and abiotic stressors (Soler *et al*. 2008; Ferrenberg & Mitton 2014), while wood plays vital roles in growth, development, storage, defence against both biotic and abiotic stresses, and maintaining an upright position (Pfanz *et al*. 2002; Abreu *et al*. 2020; Wang *et al*. 2021). Despite different functions, bark and wood also share many metabolic features with leaves, underlining the basic similarities of metabolism in all cells of the tree.

Changes in plant metabolism upon herbivory have long been recognized in trees, but these have rarely been studied in a comprehensive way. Here, we show an increase in the number and abundance of specialize metabolic features upon herbivory, along with a decrease in shared features among organs and an increase in unique features. This suggests that, following herbivory, plants likely induce a broader array of metabolites, possibly involved in signalling or defence. Such diversification may suggest a mobilization of resources to mitigate damage in leaves, as the optimal defence theory explains (Tsunoda *et al*. 2017). This finding is in line with our second hypothesis, which predicted that herbivory‐induced metabolic changes would be strongest in damaged organs, but that certain metabolites might be relocated to other organs. *P. nigra* bark exhibited a significant decrease in alpha chemodiversity, while other organs showed either an increase (young leaves, wood, leaves) or no significant change (old leaves, root). The increase in unique mass features among organs implies that each organ exhibits a unique response to herbivory that may be tailored to enhance its resistance or tolerance. This suggests that resource reallocation may be occurring, consistent with our hypothesis that plants transport key compounds to balance growth and defence. Whether or not such organ‐level differences lead to plant regrowth or increased protection from herbivory requires further study. Such differences may also convey information to herbivores regarding organ identity, physiological status, and nutritional quality that may have a large impact on plant–herbivore interactions (Leong & Last 2017; Machado *et al*. 2021; Hauri *et al*. 2022).

Our targeted analysis revealed significant organ‐specific differences in the amino acid and sugar composition of black poplar. Amino acids are essential for plant growth, development, and stress resistance in all cells (Pichersky & Lewinsohn 2011; Pratelli & Pilot 2014; Yang *et al*. 2020), as well as being precursors of numerous other metabolites, including defence compounds (Hildebrandt *et al*. 2015; Zoric *et al*. 2019). Yet notable disparities were recorded among organs, with the greatest divergence observed in the bark. Herbivory led to a significant reduction in the concentrations of proline, valine, and threonine in the bark. On the other hand, upon herbivory there was an increase in the concentration of sugars (e.g., sucrose and verbascose) in the bark. These results support our expectation that metabolic changes would be strongest in damaged organs, while also highlighting the role of the bark in redistributing resources, as predicted. These trends point to the role of bark in the redistribution of compounds to and from other organs, such as leaves and roots. Such processes could enhance resilience to herbivory, but further research is required to elucidate the precise mechanisms and benefits involved.

Targeted analysis of salicinoids, the most prevalent group of phenolic specialized metabolites in poplar species, also showed organ‐specific differences, with highest concentrations in leaves. Salicinoids play a significant role in poplar–herbivore interactions, acting as toxins and deterrents to various insect species (Boeckler *et al*. 2011, 2013, 2016; Fabisch *et al*. 2019; Movahedi *et al*. 2021). We also observed differences in the concentration of phenolics between new and old leaves, which may reflect variations in leaf value and herbivore susceptibility (Barton & Boege 2017; Albert *et al*. 2018). However, there were no significant changes in salicinoids upon herbivory. In fact, short‐term inducibility of salicinoids upon generalist insect feeding has rarely been observed (Boeckler *et al*. 2013; Fabisch *et al*. 2019). In our comparison, leaves were exposed to herbivory for only 48 h, which may not have been long enough for the induction of increased amounts of defence compounds constitutively present in high concentrations that also require *de novo* biosynthesis.

In conclusion, our results illustrate the existence of distinct organ‐specific metabolic profiles in black poplar that may reflect differences in growth and defence strategies. The metabolic changes recorded after herbivory emphasize the plasticity of poplar metabolomes and the fact that changes in central metabolism after insect feeding are at least as significant as changes in specialized metabolites. These findings highlight the role of metabolic plasticity in enabling plants to respond dynamically to herbivory and stress, and the importance of resource allocation strategies in maintaining metabolic homeostasis. Specifically, our results suggest that plants do not maintain completely distinct metabolic pools, but rather redistribute key metabolites between organs to optimize resource use while minimizing energetic costs.

## AUTHOR CONTRIBUTIONS

SY‐V, MP, J‐PS, and SBU conceived the project, and SY‐V and MP did the sampling and analyzed the data. SY‐V, MP and MR performed the chemical analyses. SY‐V, MP, J‐PS, and SBU wrote the paper. All authors reviewed and edited the manuscript.

## CONFLICT OF INTEREST STATEMENT

The authors declares no conflict of interest.

## Supporting information


**Fig. S1.** Cross‐validation of organ metabolome differences. PCA of features selected through non‐targeted analysis, showing the grouping of the samples by organs (colour) and comparison (shape). The PCA was performed using log‐transformed, centered, and Pareto‐scaled data, as described in the “Statistics analysis” section.
**Fig. S2.** Phenotypic differences between caterpillar‐infested (*n* = 12) and non‐infested (control, *n* = 12) *Populus nigra* trees. (A) Final height (cm) and (B) number of leaves in trees without herbivory (in grey) and trees subjected to herbivory by the generalist *Lymantria dispar* (in red) for 48 h. (C) Percentage leaf area loss (%) caused by caterpillar feeding in young (dark green) and old (light green) leaves of infested trees. Each boxplot incorporates the median line, interquartile range (IQR), and whiskers extending to 1.5 times the IQR. Data were analysed by Student's *t*‐test.
**Fig. S3.** Concentration of phenolic compounds in five different organs of young *Populus nigra* trees. Boxplot illustrates the concentration (mg g^−1^ DW) of (A) salicin, (B) salicortin, (C) Proanthocyanidin B1 (PAB1), (D) salicortin‐6‐benzoate, (E) homaloside D and (F) catechin across various organs in the *stem–leaf* (*n* = 12 per treatment) and the *root–leaf comparison* (lower graphs in panels A–E, *n* = 15 per treatment). Please note that catechin was not detected in the *root–leaf comparison*. The grey box plot corresponds to control plants, while the red box plot represents infested trees. The *x*‐axis denotes the concentration levels, and the *y*‐axis categorizes the organs under investigation. Each boxplot incorporates the median line, interquartile range (IQR), and whiskers extending to 1.5 times the IQR. Individual data points beyond this range are considered outliers and displayed as dots. Significance values based on a Linear Mixed Effect Model (LMER): *, *P* < 0.05; **, *P* < 0.01; ***, *P* < 0.001; n.s., not significant. See Table S4 for details on the statistical analysis.
**Fig. S4.** Sugar concentrations in four different organs of young *Populus nigra* trees. Boxplots illustrate the concentration (μg g^−1^ DW) of (A) glucose, (B) fructose, (C) mannitol, (D) sucrose, (E) raffinose, (F) stachyose, and (G) verbascose in various organs of young black poplar trees in two comparisons (for *stem–leaf comparison n* = 12 per treatment, for the *root–leaf comparison n* = 15 per treatment). Note that verbascose was not detected in the *root–leaf comparison*. The grey box plot corresponds to control plants, while the red box plot represents caterpillar‐infested trees. The *x*‐axis denotes the concentrations, and the *y*‐axis categorizes the respective organs investigated. Each boxplot incorporates the median line, interquartile range (IQR), and whiskers extending to 1.5 times the IQR. Individual data points beyond this range are considered outliers and displayed as dots. Significance values based on a Linear Mixed Effect Model (LMER): *, *P* < 0.05; **, *P* < 0.01; ***, *P* < 0.001; n.s., not significant. See Table S5 for statistical analysis.
**Fig. S5.** Comparison of organ‐specific metabolites in you *Populus nigra* trees following *Lymantria dispar* caterpillar infestation for 48 h. Illustration of Venn diagrams depicting features identified through non‐targeted metabolomics analysis for the *stem–leaf* (*n* = 12 per treatment) (A) and *root–leaf* (*n* = 15 per treatment) (B) *comparisons*. Additionally, Venn diagrams (C, D) illustrate constitutive features (in grey) of non‐damaged control trees and caterpillar‐induced features (in red) for each organ in the *stem–leaf* and *root–leaf comparisons*, respectively. The overlapping regions in the Venn diagrams denote shared metabolic features among different plant organs, offering insights into metabolic similarities across the analysed samples.
**Fig. S6.** Herbivory‐induced dynamic changes in target metabolites in young *Populus nigra* trees. Volcano plots illustrate the comparison of compounds selected through targeted analysis, showcasing the intensity alterations in the presence of herbivory for each studied organ in the *stem–leaf* (*n* = 12 per treatment) (A) and the *root–leaf comparison* (*n* = 15 per treatment) (B). Upward shifts (red dots) represent enrichment, while downward shifts (blue dots) indicate depletion in the compounds. Grey dots do not significantly change upon the treatment. The *x*‐axis represents the log_2_ fold change in relative abundance, while the *y*‐axis represents the ‐log10 *P*‐value. The horizontal dashed line indicates the significance threshold (*P*‐value = 0.05), and the vertical dashed lines indicate the fold change cutoffs (log^2^ fold change = ±1.5). Features above the horizontal line and beyond the vertical lines are considered significantly differentially expressed.
**Fig. S7.** Boxplots of Hill Shannon's diversity in young *Populus nigra* organs after *Lymantria dispar* caterpillar infestation for 48 h. (A) *Stem–leaf* (*n* = 12 per treatment) and (B) *root–leaf comparisons* (*n* = 15 per treatment) based on non‐targeted data analysis. The *x*‐axis represents Shannon's diversity, while the *y*‐axis categorizes the organs. Boxplots without lines depict control samples, whereas those with diagonal lines represent infested trees. Each boxplot displays the median line, interquartile range (IQR), and whiskers extending to 1.5 times the IQR. Outliers beyond this range are denoted as black dots. Significance values based on a Linear Mixed Effect Model (LMER) (Table S7) followed by post‐hoc pairwise comparisons to assess pairwise comparisons between organs and treatments: *, *P* < 0.05; **, *P* < 0.01; ***, *P* < 0.001.
**Table S1.** Internal standards used for non‐targeted metabolomics analysis of different organs of young *Populus nigra* trees.
**Table S2.** Parameters used in LC–MS/MS analysis of amino acids by LC–MS/MS on a triple quadrupole instrument (HPLC 1260‐QTRAP6500) in the positive ionization mode. Abbreviations: Q1, quadrupole 1; Q3, quadrupole 3; RT, retention time; DP, declustering potential; CE, collision energy.
**Table S3.** Parameters used in LC–MS/MS analysis of sugars by LC–MS/MS on a triple quadrupole instrument (HPLC 1260‐API3200) in the negative ionization mode. Abbreviations: Q1, quadrupole 1; Q3, quadrupole 3; RT, retention time; DP, declustering potential; CE, collision energy.
**Table S4.** Summary of Linear Mixed‐Effects Model (LMER) analysis testing for effects of organ, treatment, and their interaction on the concentration of phenolic compounds in *Populus nigra* leaves.
**Table S5.** Summary of Linear Mixed‐Effects Model (LMER) analysis testing for effects of organ, treatment, and their interaction on the concentration of sugars in *Populus nigra* leaves.
**Table S6.** Summary of Linear Mixed‐Effects Model (LMER) analysis testing for effects of organ, treatment, and their interaction on the concentration of amino acids in *Populus nigra* leaves.
**Table S7.** Summary of Linear Mixed‐Effects Model (LMER) analysis testing for effects of organ, treatment, and their interaction on the Hill Shannon's diversity value for *Populus nigra* leaves.

## Data Availability

After publication in Plant Biology, all data will be made available in a public repository.
