# Supplementary material for: Deciphering organ‐specific chemical changes following insect herbivory in Populus nigra using comparative metabolomics
Source: Plant Biol (Stuttg). 2025 May 28;27(5):834–46. doi: 10.1111/plb.70032 (PMC12255289; doi:10.1111/plb.70032)
Supplement: Supplementary file 1 — Fig. S1. Cross‐validation of organ metabolome differences. PCA of features selected through non‐targeted analysis, showing the grouping of the samples by organs (colour) and comparison (shape). The PCA was performed using log‐transformed, centered, and Pareto‐scaled data, as described in the “Statistics analysis” section. Fig. S2. Phenotypic differences between caterpillar‐infested (n = 12) and non‐infested (control, n = 12) Populus nigra trees. (A) Final height (cm) and (B) number of leaves in trees without herbivory (in grey) and trees subjected to herbivory by the generalist Lymantria dispar (in red) for 48 h. (C) Percentage leaf area loss (%) caused by caterpillar feeding in young (dark green) and old (light green) leaves of infested trees. Each boxplot incorporates the median line, interquartile range (IQR), and whiskers extending to 1.5 times the IQR. Data were analysed by Student's t‐test. Fig. S3. Concentration of phenolic compounds in five different organs of young Populus nigra trees. Boxplot illustrates the concentration (mg g−1 DW) of (A) salicin, (B) salicortin, (C) Proanthocyanidin B1 (PAB1), (D) salicortin‐6‐benzoate, (E) homaloside D and (F) catechin across various organs in the stem–leaf (n = 12 per treatment) and the root–leaf comparison (lower graphs in panels A–E, n = 15 per treatment). Please note that catechin was not detected in the root–leaf comparison. The grey box plot corresponds to control plants, while the red box plot represents infested trees. The x‐axis denotes the concentration levels, and the y‐axis categorizes the organs under investigation. Each boxplot incorporates the median line, interquartile range (IQR), and whiskers extending to 1.5 times the IQR. Individual data points beyond this range are considered outliers and displayed as dots. Significance values based on a Linear Mixed Effect Model (LMER): *, P < 0.05; **, P < 0.01; ***, P < 0.001; n.s., not significant. See Table S4 for details on the statistical analysis. Fig. S4. [file PLB-27-834-s001.docx]

**Supplemental Material**

**Deciphering organ-specific chemical changes following insect herbivory in *Populus nigra* using comparative metabolomics**

Sol Yepes-Vivas^1ⱡ^, Moritz Popp^2ⱡ^, Michael Reichelt^1^, Jonathan Gershenzon^1^, Jorg-Peter Schnitzler^2*^, & Sybille B. Unsicker^1,3*^.

^1^Department of Biochemistry, Max Planck Institute for Chemical Ecology, Hans-Knöll Str. 8, 07745 Jena, Germany

^2^Research Unit Environmental Simulation, Helmholtz Zentrum München, Ingolstädter Landstraße 1, 85764 Oberschleißheim, Germany

^3^Plant-Environment-Interactions Group, Botanical Institute, Christian-Albrechts-Universität zu Kiel, Am Botanischen Garten 5, 24118 Kiel, Germany

ⱡ Both authors contributed equally and share the first authorship

***Corresponding authors:** Sybille B. Unsicker^3^ [sunsicker@bot.uni-kiel.de](mailto:sunsicker@bot.uni-kiel.de), Jörg-Peter Schnitzler^2^ [joergpeter.schnitzler@helmholtz-munich.de](mailto:joergpeter.schnitzler@helmholtz-munich.de)

**SUPPLEMENTAL FIGURES**


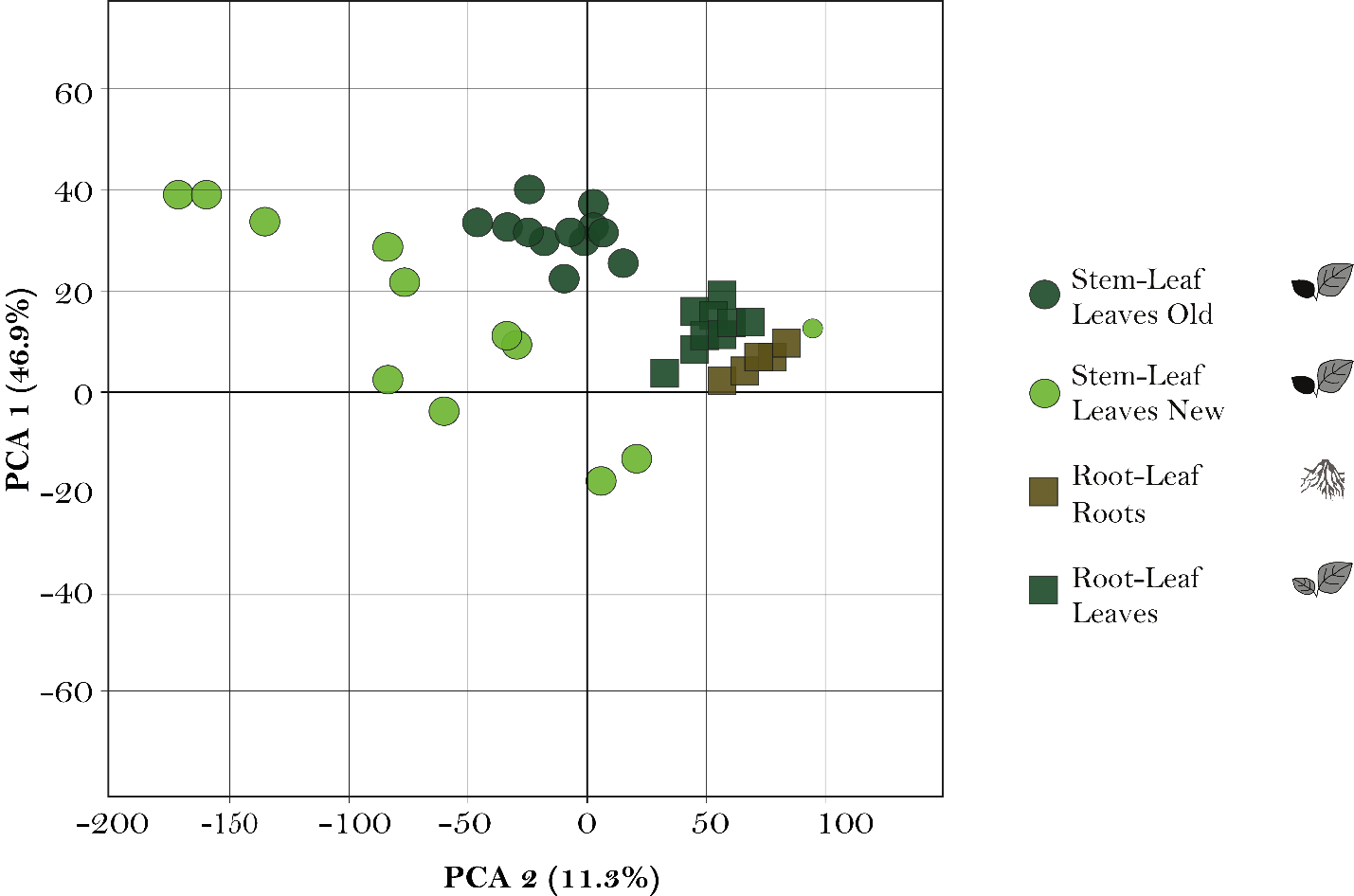


**Figure S1.** Cross-validation of organ metabolome differences. Principal Component Analysis (PCA) of features selected through non-targeted analysis, showing the grouping of the samples by organs (color) and comparison (shape). The PCA was performed using log-transformed, centered, and Pareto-scaled data, as described in the “Statistics analysis” section

**
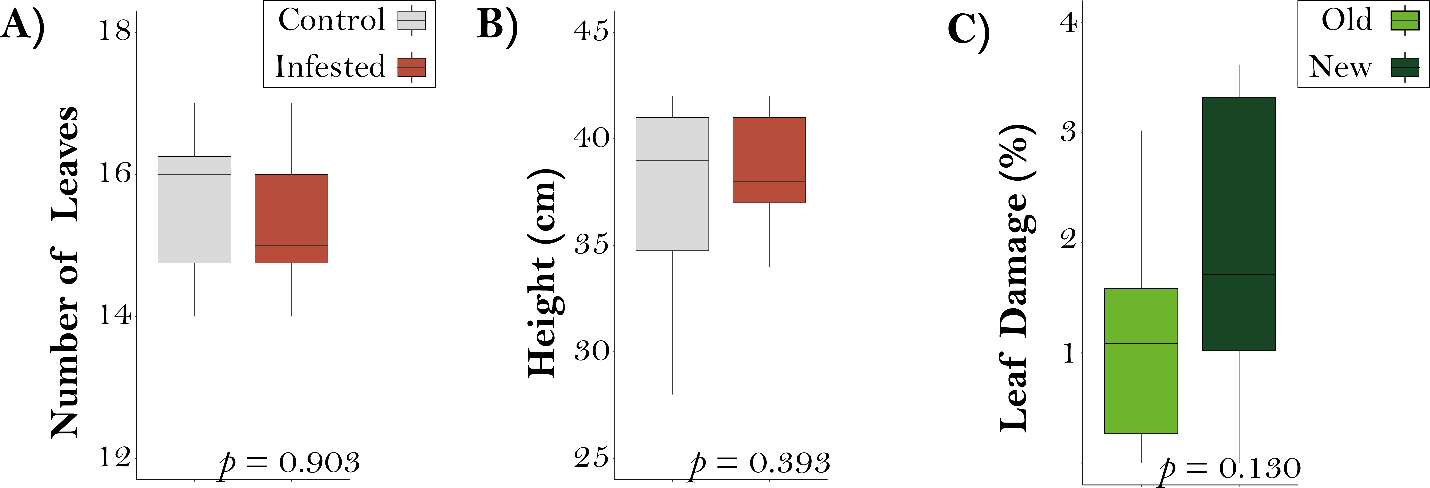
**

**Figure S2.** Phenotypic differences between caterpillar-infested (n=12) and non-infested (control, n = 12) *Populus nigra* trees. (A) number of leaves and (B) final height (cm) in trees without herbivory (in gray) and trees subjected to herbivory by the generalist *Lymantria dispar* (in red) for 48 hours. (C) Percent leaf area loss (%) caused by caterpillar feeding in young (dark green) and old leaves (light green) of infested trees. Each boxplot incorporates the median line, interquartile range (IQR), and whiskers extending to 1.5 times the IQR. Data were analyzed by Student’s t-test.

**
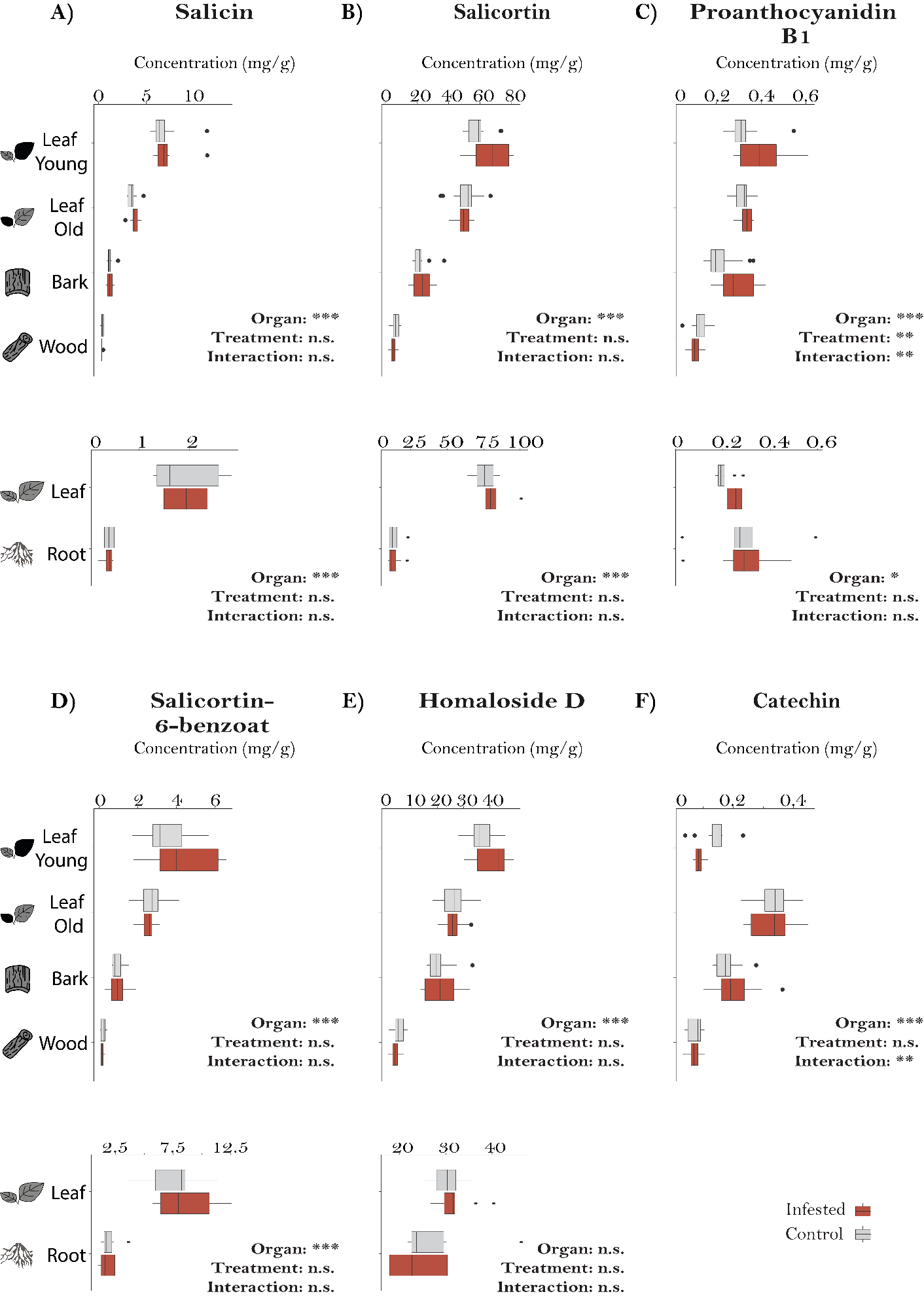
**

**Figure S3.** Concentration of phenolic compounds in five different organs of young *Populus nigra* trees. Boxplot illustrates the concentration (mg/g DW) of (A) salicin, (B) salicortin, (C) Proanthocyanidin B1 (PAB1), (D) salicortin-6-benzoate, (E) homaloside D and, (F) catechin across various organs in the *stem-leaf* (n = 12 per treatment) and the *root-leaf comparison* (lower graphs in panels A-E, n=15 per treatment). Please note that catechin was not detected in the *root-leaf comparison*. The gray box plot corresponds to control plants, while the red box plot represents infested trees. The x-axis denotes the concentration levels, and the y-axis categorizes the organs under investigation. Each boxplot incorporates the median line, interquartile range (IQR), and whiskers extending to 1.5 times the IQR. Individual data points beyond this range are considered outliers and displayed as dots. Significance values based on a Linear Mixed Effect Model (LMER): *, p < 0.05; **, p < 0.01; ***, p < 0.001; n.s., not significant. See Table S4 for details on the statistical analysis.

**
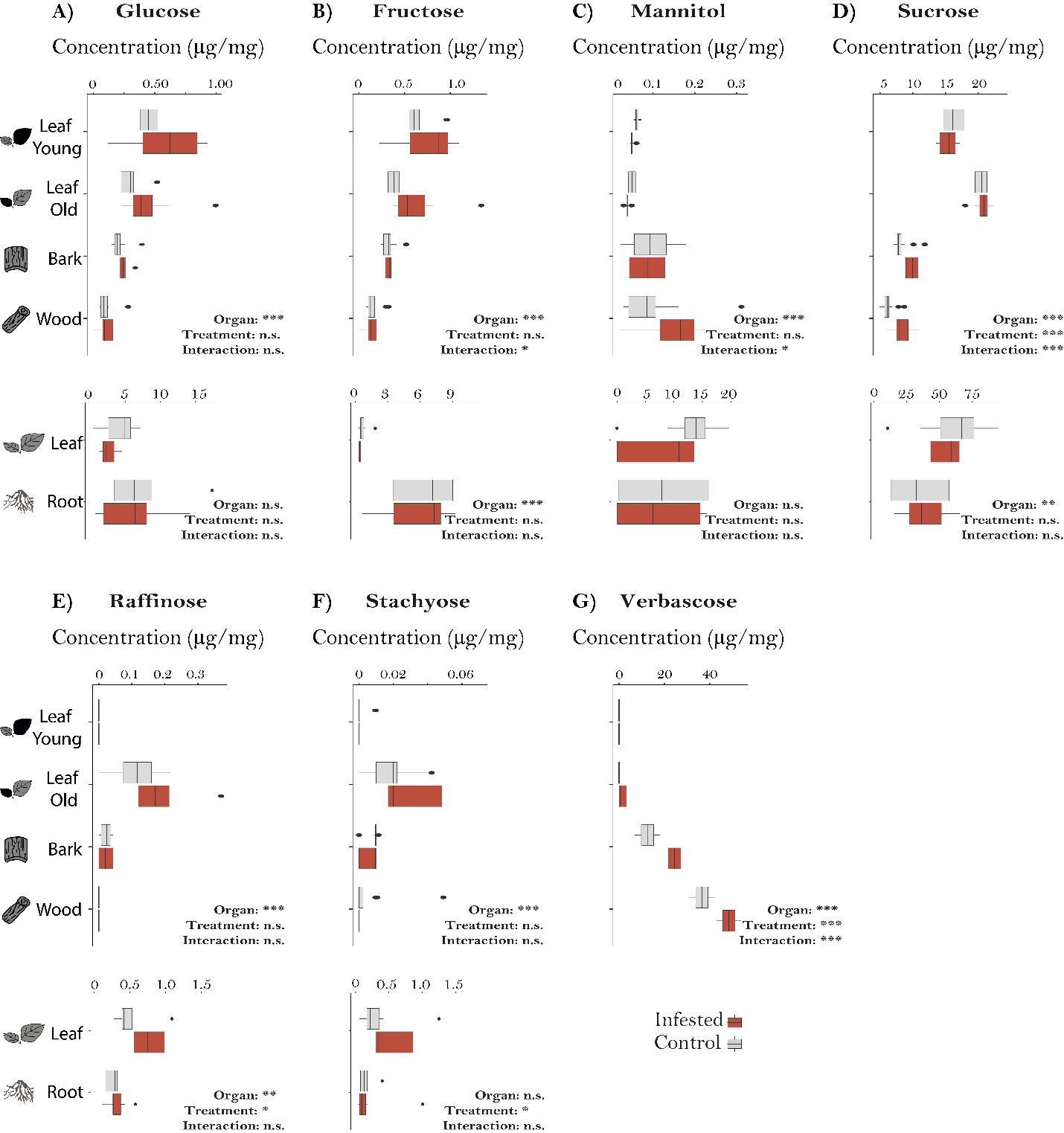
**

**Figure S4.** Sugar concentrations in four different organs of young *Populus nigra* trees. Boxplots illustrate the concentration (ug/g DW) of (A) glucose, (B) fructose, (C) mannitol, (D) sucrose, (E) raffinose, (F) stachyose, and (G) verbascose various organs in young black poplar trees in two comparisons (for *steam-leaf comparison* n = 12 per treatment, for the *root-leaf comparison* n=15 per treatment). Note that verbascose was not detected in the *root-leaf comparison*. The gray box plot corresponds to control plants, while the red box plot represents caterpillar-infested trees. The x-axis denotes the concentrations, and the y-axis categorizes the respective organs investigated. Each boxplot incorporates the median line, interquartile range (IQR), and whiskers extending to 1.5 times the IQR. Individual data points beyond this range are considered outliers and displayed as dots. Significance values based on a Linear Mixed Effect Model (LMER): *, p < 0.05; **, p < 0.01; ***, p < 0.001; n.s., not significant. See Table S5 for statistical analysis.

**
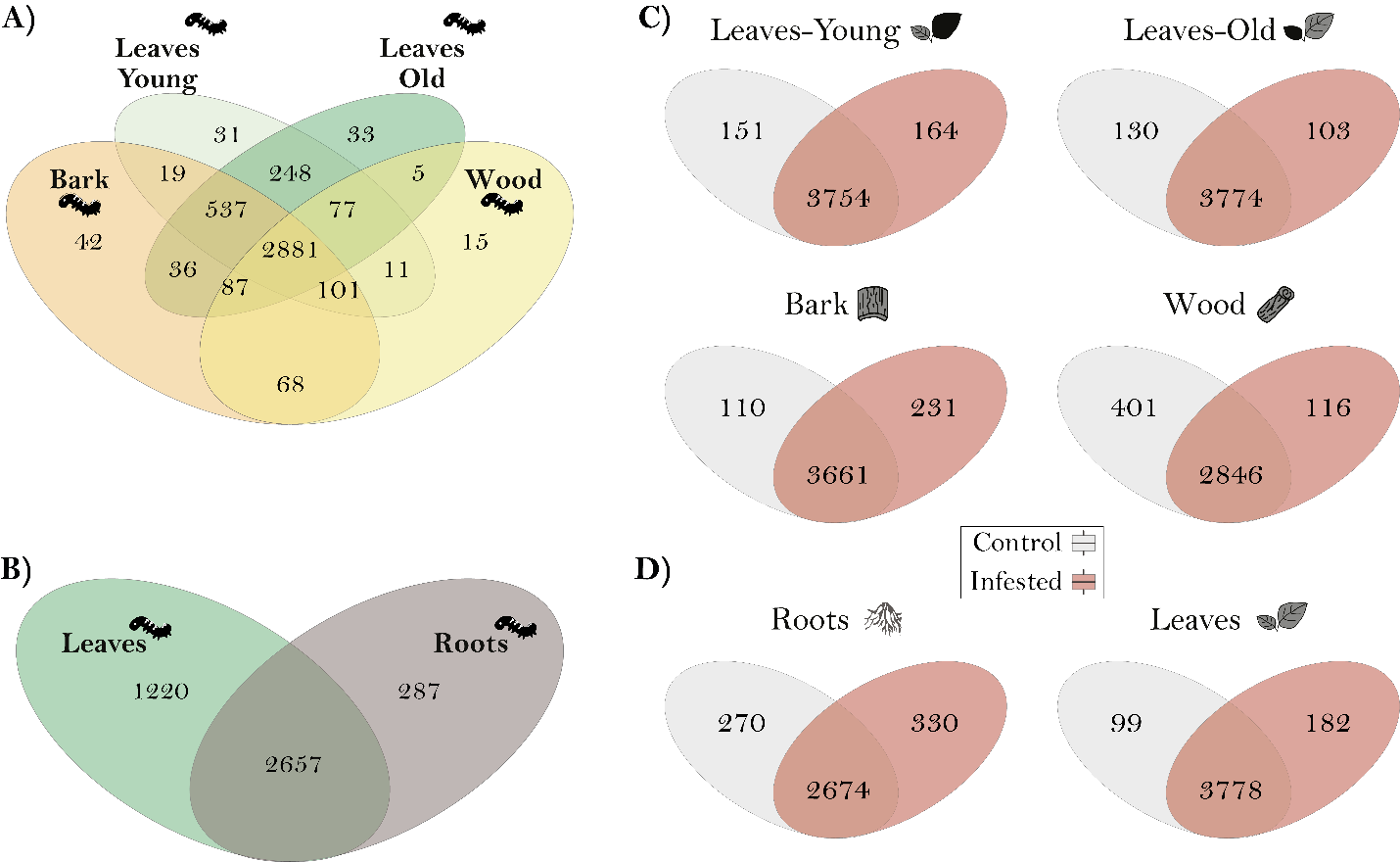
**

**Figure S5.** Comparison of organ-specific metabolites in you *Populus nigra* trees following *Lymantria dispar* caterpillar infestation for 48 hours. Illustration of Venn diagrams depicting features identified through non-targeted metabolomics analysis for the *stem-leaf* (n = 12 per treatment) (A) and *root-leaf* (n=15 per treatment) (B) *comparisons*. Additionally, Venn diagrams (C, D) illustrate constitutive features (in gray) of non-damaged control trees and caterpillar-induced features (in red) for each organ in the *stem-leaf* and *root-leaf comparisons*, respectively. The overlapping regions in the Venn diagrams denote shared metabolic features among different plant organs, offering insights into metabolic similarities across the analyzed samples.

**
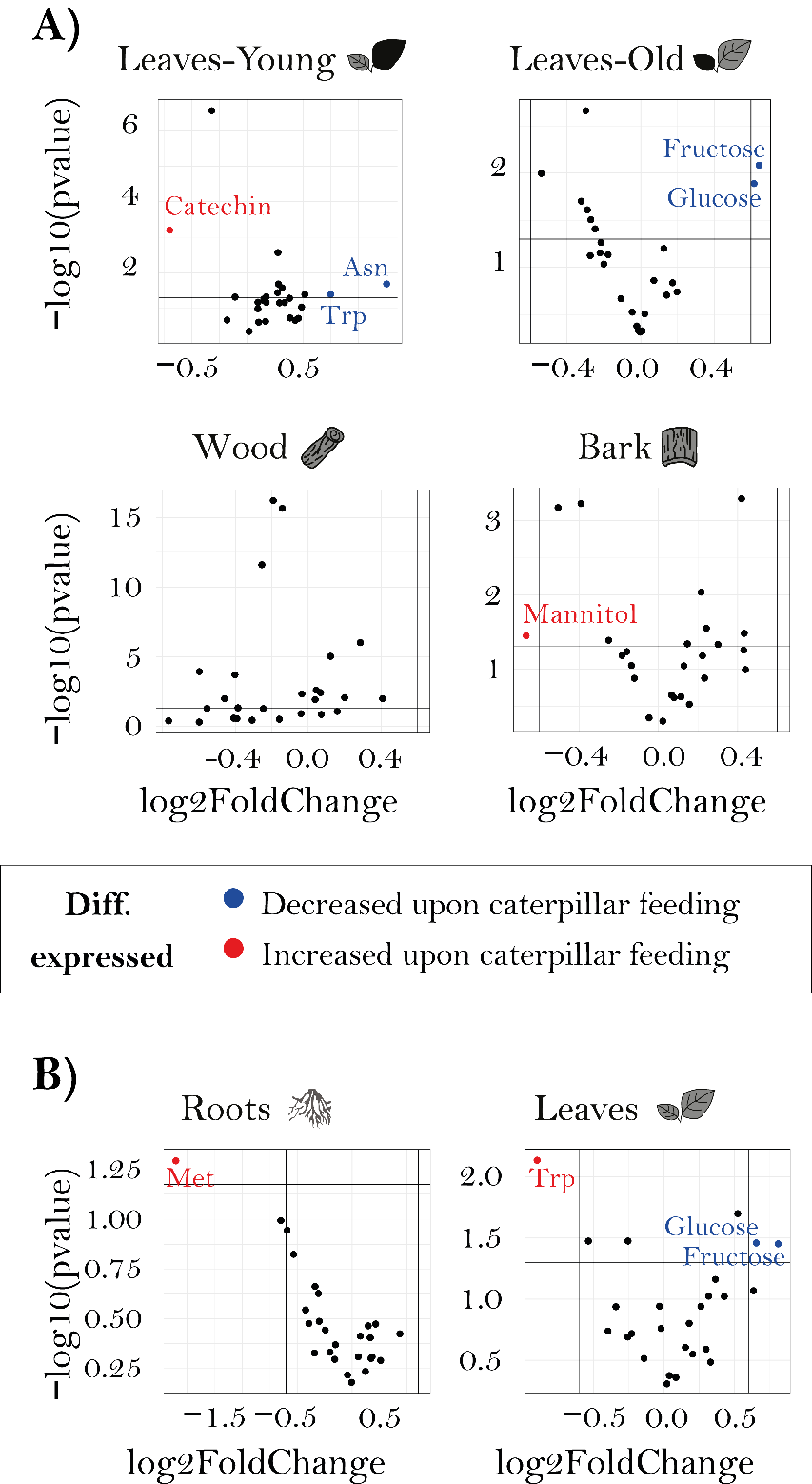
**

**Figure S6.** Herbivory-induced dynamic changes in target metabolites in young *Populus nigra* trees. Volcano plots illustrate the comparison of compounds selected through targeted analysis, showcasing the intensity alterations in the presence of herbivory for each studied organ in the *stem-leaf* (n=12 per treatment) (A) and the *root-leaf* *comparison* (n = 15 per treatment) (B). Upward shifts (red dots) represent enrichment, while downward shifts (blue dots) indicate depletion in the compounds. Grey dots do not significantly change upon the treatment. The x-axis represents the log_2_ fold change in relative abundance, while the y-axis represents the -log10 p-value. The horizontal dashed line indicates the significance threshold (p-value = 0.05), and the vertical dashed lines indicate the fold change cutoffs (log_2_ fold change = ±1.5). Features above the horizontal line and beyond the vertical lines are considered significantly differentially expressed.

**
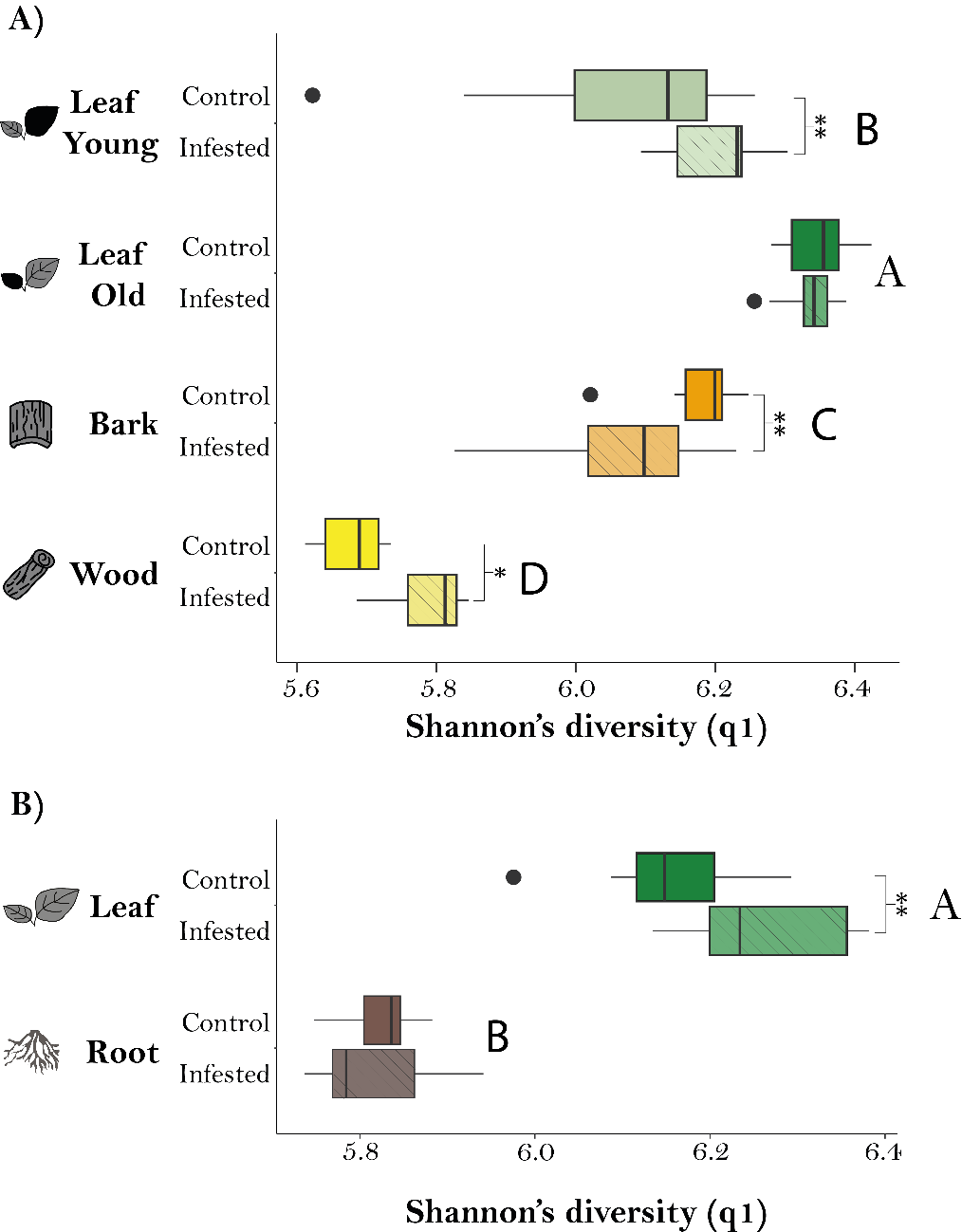
**

**Figure S7.** Boxplots of Hill Shannon’s diversity in young *Populus nigra* organs after *Lymantria dispar* caterpillar infestation for 48 hours. (A) *Stem-leaf* (n=12 per treatment) and (B) *root-leaf comparisons* (n = 15 per treatment) based on non-targeted data analysis. The x-axis represents Shannon’s diversity, while the y-axis categorizes the organs. Boxplots without lines depict control samples, whereas those with diagonal lines represent infested trees. Each boxplot displays the median line, interquartile range (IQR), and whiskers extending to 1.5 times the IQR. Outliers beyond this range are denoted as black dots. Significance values on a Linear Mixed-Effects Model (LMER) (Table S7). followed by post-hoc pairwise comparisons to assess pairwise comparisons between organs and treatments *, p < 0.05; **, p < 0.01; ***, p < 0.001.

**SUPPLEMENTAL TABLES**

**Table S1.** Internal standards used for non-targeted metabolomics analysis of different organs of young *Populus nigra* trees.

| **Compound** | **concentration [µg/ml]** |
| --- | --- |
| Magnolol | 0.054 |
| Camphorsulfonic acid | 0.047 |
| 3,4-Dihydroxymandelic acid | 0.037 |
| Plumbagin | 0.038 |
| 3',4'-dihydroxyacetophenone | 0.031 |
| Xanthotoxin | 0.043 |
| Coniferin | 0.069 |
| Rosmarinic acid | 0.072 |

**Table S2.** Parameters used in LC-MS/MS analysis of amino acids by LC-MS/MS on a triple quadrupole instrument (HPLC 1260 (Agilent Technologies)-QTRAP6500 (SCIEX)) in the positive ionization mode. Abbreviations: Q1, quadrupole 1; Q3, quadrupole 3; RT, retention time; DP, declustering potential; CE, collision energy.

| **Compound** | **Q1** | **Q3** | **RT (min)** | **Internal standard** | **IS Q1** | **IS Q3** | **DP** | **CE** |
| --- | --- | --- | --- | --- | --- | --- | --- | --- |
| Ala | 90.1 | 44.1 | 0.5 | 13C,15N-Ala | 94.1 | 47.1 | 20 | 17 |
| Ser | 106.0 | 60.1 | 0.5 | 13C,15N-Ser | 110.0 | 63.1 | 20 | 15 |
| Pro | 116.1 | 70 | 0.5 | 13C,15N-Pro | 122.1 | 75.0 | 20 | 19 |
| Val | 118.1 | 72.2 | 0.5 | 13C,15N-Val | 124.1 | 77.2 | 20 | 13 |
| Thr | 120.1 | 74.2 | 0.5 | 13C,15N-Thr | 125.1 | 78.2 | 20 | 13 |
| Ile | 132.2 | 86.1 | 1.1 | 13C,15N-Ile | 139.2 | 92.1 | 20 | 13 |
| Leu | 132.2 | 86.1 | 1.3 | 13C,15N-Leu | 139.2 | 92.1 | 20 | 13 |
| Asp | 134.1 | 74.1 | 0.5 | 13C,15N-Asp | 139.1 | 77.1 | 20 | 19 |
| Glu | 148.1 | 102.1 | 0.5 | 13C,15N-Glu | 154.1 | 107.1 | 20 | 15 |
| Met | 150.2 | 104.1 | 0.7 | 13C,15N-Met | 156.2 | 109.1 | 20 | 13 |
| His | 156.2 | 110.1 | 0.4 | 13C,15N-His | 165.2 | 118.1 | 20 | 17 |
| Phe | 166.2 | 120.2 | 2.6 | 13C,15N-Phe | 176.2 | 129.2 | 20 | 17 |
| Arg | 175.1 | 70.1 | 0.4 | 13C,15N-Arg | 185.1 | 75.1 | 20 | 31 |
| Tyr | 182.1 | 136.2 | 1.4 | 13C,15N-Tyr | 192.1 | 145.2 | 20 | 17 |
| Asn | 133.1 | 74.1 | 0.5 | 13C,15N-Asp |  |  | 20 | 21 |
| Gln | 147.1 | 130 | 0.5 | 13C,15N-Gln | 154.1 | 136.0 | 20 | 13 |
| Trp | 205.2 | 188.1 | 3.2 | D5-Trp | 210.0 | 193.0 | 20 | 13 |

**Table S3.** Parameters used in LC-MS/MS analysis of sugars by LC-MS/MS on a triple quadrupole instrument (HPLC 1260 (Agilent Technologies)- API3200 (AB SCIEX)) in the negative ionization mode. Abbreviations: Q1, quadrupole 1; Q3, quadrupole 3; RT, retention time; DP, declustering potential; CE, collision energy.

| **Q1** | **Q3** | **RT (min)** | **Compound** | **DP** | **CE** |
| --- | --- | --- | --- | --- | --- |
| 178.8 | 89.0 | 6.7 | Glucose | -25 | -10 |
| 178.801 | 89.0 | 5.6 | Fructose | -25 | -12 |
| 340.9 | 59.0 | 8.2 | Sucrose | -45 | -46 |
| 181.0 | 89.0 | 6.3 | Mannitol | -35 | -22 |
|  |  |  | Raffinose |  |  |
|  |  | 11.8 | Stachyose |  |  |
|  |  | 10.2 | Verbascose |  |  |
| 185.0 | 92.0 | 6.7 | 13-C6-Glucose | -25 | -10 |
| 185.01 | 92.0 | 5.6 | 13-C6-Fructose | -25 | -12 |

**Table S4.** Summary of Linear Mixed Effect Model (LMER) analysis testing for effects of organ, treatment, and their interaction on the concentration of phenolic compounds in *Populus nigra* leaves**.**

|  | ***Steam-Leaf Comparison*** | | | | | | | | |
| --- | --- | --- | --- | --- | --- | --- | --- | --- | --- |
|  | **Organ** | | | **Treatment** | | | **Organ*treatment** | | |
|  | **Df** | **Chisq** | ***p*** | **Df** | **Chisq** | **p** | **Df** | **Chisq** | ***p*** |
| Salicin | 3 | 778.601 | **<0.001** | 1 | 0.034 | 0.854 | 3 | 2.861 | 0.414 |
| Salicortin | 3 | 654.241 | **<0.001** | 1 | 0.162 | 0.688 | 3 | 6.536 | 0.088 |
| PA B1 | 3 | 93.940 | **<0.001** | 1 | 8.310 | **0.004** | 3 | 11.477 | **0.009** |
| Salicortin-6-benzoat | 3 | 265.785 | **<0.001** | 1 | 0.080 | 0.778 | 3 | 5.444 | 0.142 |
| Homaloside D | 3 | 356.612 | **<0.001** | 1 | 0.078 | 0.781 | 3 | 4.241 | 0.237 |
| Catechin | 3 | 179.672 | **<0.001** | 1 | 1.226 | 0.268 | 3 | 10.443 | **0.015** |
|  | ***Root-Leaf Comparison*** | | | | | | | | |
|  | **Organ** | | | **Treatment** | | | **Organ*treatment** | | |
|  | **Df** | **Chisq** | ***p*** | **Df** | **Chisq** | **p** | **Df** | **Chisq** | ***p*** |
| Salicin | 1 | 68.026 | **<0.001** | 1 | 0.022 | 0.881 | 1 | 0.019 | 0.890 |
| Salicortin | 1 | 469.588 | **<0.001** | 1 | 0.564 | 0.453 | 1 | 0.409 | 0.523 |
| PA B1 | 1 | 4.911 | **0.027** | 1 | 1.653 | 0.199 | 1 | 0.645 | 0.422 |
| Salicortin-6-benzoat | 1 | 70.945 | **<0.001** | 1 | 1.432 | 0.231 | 1 | 0.907 | 0.341 |
| Homaloside D | 1 | 2.007 | 0.157 | 1 | 0.314 | 0.575 | 1 | 0.673 | 0.412 |

**Table S5.** Summary of Linear Mixed Effect Model (LMER) analysis testing for effects of organ, treatment, and their interaction on the concentration of sugars in *Populus nigra* leaves.

|  | ***Steam-Leaf Comparison*** | | | | | | | | |
| --- | --- | --- | --- | --- | --- | --- | --- | --- | --- |
|  | **Organ** | | | **Treatment** | | | **Organ*treatment** | | |
|  | **Df** | **Chisq** | ***P*** | **Df** | **Chisq** | ***P*** | **Df** | **Chisq** | ***p*** |
| Glucose | 3 | 73.438 | **<0.001** | 1 | 0.381 | 0.537 | 3 | 4.925 | 0.177 |
| Fructose | 3 | 81.865 | **<0.001** | 1 | 0.018 | 0.892 | 3 | 9.397 | **0.024** |
| Mannitol | 3 | 11.031 | **0.012** | 1 | 0.291 | 0.590 | 3 | 10.777 | **0.013** |
| Sucrose | 3 | 893.287 | **<0.001** | 1 | 14.999 | **<0.001** | 3 | 27.765 | **<0.001** |
| Raffinose | 3 | 149.637 | **<0.001** | 1 | 0.083 | 0.773 | 3 | 6.988 | 0.072 |
| Stachyose | 3 | 42.211 | **<0.001** | 1 | 1.287 | 0.257 | 3 | 5.421 | 0.143 |
| Verbascose | 3 | 2546.176 | **<0.001** | 1 | 101.351 | **<0.001** | 3 | 52.560 | **<0.001** |
|  | ***Root-Leaf Comparison*** | | | | | | | | |
|  | **Organ** | | | **Treatment** | | | **Organ*treatment** | | |
|  | **Df** | **Chisq** | ***P*** | **Df** | **Chisq** | ***P*** | **Df** | **Chisq** | ***P*** |
| Glucose | 1 | 0.713 | 0.398 | 1 | 1.208 | 0.272 | 1 | 0.431 | 0.511 |
| Fructose | 1 | 29.652 | **<0.001** | 1 | 0.401 | 0.527 | 1 | 0.202 | 0.653 |
| Mannitol | 1 | 1.627 | 0.202 | 1 | 2.486 | 0.115 | 1 | 0.605 | 0.437 |
| Sucrose | 1 | 6.783 | **0.009** | 1 | 0.047 | 0.829 | 1 | 0.208 | 0.648 |
| Raffinose | 1 | 7.678 | **0.006** | 1 | 3.991 | **0.046** | 1 | 0.528 | 0.467 |
| Stachyose | 1 | 2.614 | 0.106 | 1 | 4.224 | **0.040** | 1 | 1.403 | 0.236 |

**Table S6.** Summary of Linear Mixed Effect Model (LMER) analysis testing for effects of organ, treatment, and their interaction on the concentration of amino acids in *Populus nigra* leaves.

|  | ***Steam-Leaf Comparison*** | | | | | | | | |
| --- | --- | --- | --- | --- | --- | --- | --- | --- | --- |
|  | **Organ** | | | **Treatment** | | | **Organ*treatment** | | |
|  | **Df** | **Chisq** | ***p*** | **Df** | **Chisq** | ***p*** | **Df** | **Chisq** | ***p*** |
| Alanine (Ala) | 3 | 36.341 | **<0.001** | 1 | 0.358 | 0.550 | 3 | 0.345 | 0.951 |
| Aspartic Acid (Asp) | 3 | 32.763 | **<0.001** | 1 | 2.029 | 0.154 | 3 | 5.886 | 0.117 |
| Asparagine (Asn) | 3 | 244.391 | **<0.001** | 1 | 0.219 | 0.640 | 3 | 15.737 | **0.001** |
| Glutamic Acid (Glu) | 3 | 474.755 | **<0.001** | 1 | 0.782 | 0.377 | 3 | 6.603 | 0.086 |
| Glutamine (Gln) | 3 | 1000.942 | **<0.001** | 1 | 0.408 | 0.523 | 3 | 9.598 | **0.022** |
| Histidine (His) | 3 | 34.886 | **<0.001** | 1 | 3.838 | 0.050 | 3 | 12.685 | **0.005** |
| Isoleucine (Ile) | 3 | 109.114 | **<0.001** | 1 | 5.840 | **0.016** | 3 | 9.477 | **0.024** |
| Leucine (Leu) | 3 | 75.231 | **<0.001** | 1 | 9.231 | **0.002** | 3 | 15.748 | **0.001** |
| Methionine (Met) | 3 | 177.115 | **<0.001** | 1 | 1.190 | 0.275 | 3 | 5.334 | 0.149 |
| Phenylalanine (Phe) | 3 | 126.361 | **<0.001** | 1 | 3.066 | 0.080 | 3 | 8.420 | **0.038** |
| Proline (Pro) | 3 | 20.588 | **<0.001** | 1 | 8.930 | **0.003** | 3 | 12.029 | **0.007** |
| Serine (Ser) | 3 | 52.009 | **<0.001** | 1 | 7.337 | **0.007** | 3 | 3.656 | 0.301 |
| Threonine (Thr) | 3 | 122.197 | **<0.001** | 1 | 20.513 | **<0.001** | 3 | 24.112 | **<0.001** |
| Tryptophan (Trp) | 3 | 50.877 | **<0.001** | 1 | 2.823 | 0.093 | 3 | 15.980 | **0.001** |
| Tyrosine (Tyr) | 3 | 86.478 | **<0.001** | 1 | 7.094 | **0.008** | 3 | 11.045 | **0.011** |
| Valine (Val) | 3 | 237.991 | **<0.001** | 1 | 13.839 | **<0.001** | 3 | 17.169 | **0.001** |
|  | ***Root-Leaf Comparison*** | | | | | | | | |
|  | **Organ** | | | **Treatment** | | | **Organ*treatment** | | |
|  | **Df** | **Chisq** | ***p*** | **Df** | **Chisq** | ***p*** | **Df** | **Chisq** | ***p*** |
| Alanine (Ala) | 1 | 3.362 | 0.067 | 1 | 0.137 | 0.711 | 1 | 0.058 | 0.810 |
| Arginine (Arg) | 1 | 8.603 | **0.003** | 1 | 0.190 | 0.663 | 1 | 0.058 | 0.809 |
| Aspartic Acid (Asp) | 1 | 8.096 | **0.004** | 1 | 0.528 | 0.468 | 1 | 0.110 | 0.740 |
| Asparagine (Asn) | 1 | 36.701 | **<0.001** | 1 | 0.017 | 0.896 | 1 | 0.019 | 0.889 |
| Glutamic Acid (Glu) | 1 | 8.357 | **0.004** | 1 | 1.055 | 0.304 | 1 | 0.905 | 0.341 |
| Glutamine (Gln) | 1 | 37.081 | **<0.001** | 1 | 0.078 | 0.779 | 1 | 0.152 | 0.697 |
| Glycine (Gly) | 1 | 7.203 | **0.007** | 1 | 0.650 | 0.420 | 1 | 2.454 | 0.117 |
| Histidine (His) | 1 | 3.853 | **0.050** | 1 | 0.004 | 0.949 | 1 | 0.009 | 0.924 |
| Isoleucine (Ile) | 1 | 7.159 | **0.007** | 1 | 0.114 | 0.736 | 1 | 2.168 | 0.141 |
| Leucine (Leu) | 1 | 0.001 | 0.978 | 1 | 1.179 | 0.278 | 1 | 0.682 | 0.409 |
| Lysine (Lys) | 1 | 17.122 | **<0.001** | 1 | 0.183 | 0.669 | 1 | 0.228 | 0.633 |
| Methionine (Met) | 1 | 10.383 | **0.001** | 1 | 1.053 | 0.305 | 1 | 5.911 | **0.015** |
| Phenylalanine (Phe) | 1 | 6.629 | **0.010** | 1 | 3.032 | 0.082 | 1 | 4.478 | **0.034** |
| Proline (Pro) | 1 | 1.858 | 0.173 | 1 | 0.873 | 0.350 | 1 | 0.108 | 0.743 |
| Serine (Ser) | 1 | 6.393 | **0.011** | 1 | 1.112 | 0.292 | 1 | 1.824 | 0.177 |
| Threonine (Thr) | 1 | 0.740 | 0.390 | 1 | 4.598 | 0.032 | 1 | 4.772 | **0.029** |
| Tryptophan (Trp) | 1 | 6.776 | **0.009** | 1 | 6.831 | **0.009** | 1 | 0.896 | 0.344 |
| Tyrosine (Tyr) | 1 | 7.913 | **0.005** | 1 | 3.726 | 0.054 | 1 | 5.999 | **0.014** |
| Valine (Val) | 1 | 9.010 | **0.003** | 1 | 0.146 | 0.702 | 1 | 0.461 | 0.497 |

**Table S7.** Summary of a Linear Mixed Effect Model (LMER) analysis testing for effects of organ, treatment, and their interaction on the Hill Shannon´s diversity value for *Populus nigra* leaves **#**

| ***Steam-Leaf Comparison*** | | | | | | | | | |
| --- | --- | --- | --- | --- | --- | --- | --- | --- | --- |
|  | **Organ** | | | **Treatment** | | | **Organ*Treatment** | | |
|  | **Df** | **Chisq** | ***p*** | **Df** | **Chisq** | ***p*** | **Df** | **Chisq** | ***p*** |
| Shannon´s Diversity | 3 | 111.603 | **<0.001** | 1 | 2.444 | 0.123 | 3 | 7.469 | **<0.001** |
| ***Root-Leaf Comparison*** | | | | | | | | | |
|  | **Organ** | | | **Treatment** | | | **Organ*Treatment** | | |
|  | **Df** | **Chisq** | ***p*** | **Df** | **Chisq** | ***p*** | **Df** | **Chisq** | ***p*** |
| Shannon´s Diversity | 3 | 240.745 | **<0.001** | 1 | 3.732 | 0.067 | 3 | 4.944 | 0.039 |
